# Visible-light photoredox catalysis enabled bromination of phenols and alkenes

**DOI:** 10.3762/bjoc.10.53

**Published:** 2014-03-07

**Authors:** Yating Zhao, Zhe Li, Chao Yang, Run Lin, Wujiong Xia

**Affiliations:** 1 State Key Lab of Urban Water Resource and Environment, The Academy of Fundamental and Interdisciplinary Sciences, Harbin Institute of Technology, Harbin 150080, China

**Keywords:** alkenes, bromination, phenols, photoredox catalyst, visible light

## Abstract

A mild and efficient methodology for the bromination of phenols and alkenes has been developed utilizing visible light-induced photoredox catalysis. The bromine was generated in situ from the oxidation of Br^−^ by Ru(bpy)_3_^3+^, both of which resulted from the oxidative quenching process.

## Introduction

Bromophenols serve as important synthetic intermediates for a variety of naturally occurring biologically active compounds and are also important constituents of industrial chemicals [1–5]. Thus, numerous methods were developed for the electrophilic bromination of phenols. The typical approaches include direct electrophilic halogenation by using molecular bromine or *N*-bromosuccinimide (NBS) [6–8], organometallic catalyst-promoted bromination [9–12], and the oxidative bromination of phenols [13–15]. Nevertheless, most of the methods suffer from several drawbacks such as toxic reagents, harsh conditions, low yields, and low chemo- and regioselectivity. Hence, the development of an environmentally friendly methodology for the bromination of phenols with high chemoselectivity under mild and operationally simple conditions is still appealing.

**Scheme 1:**
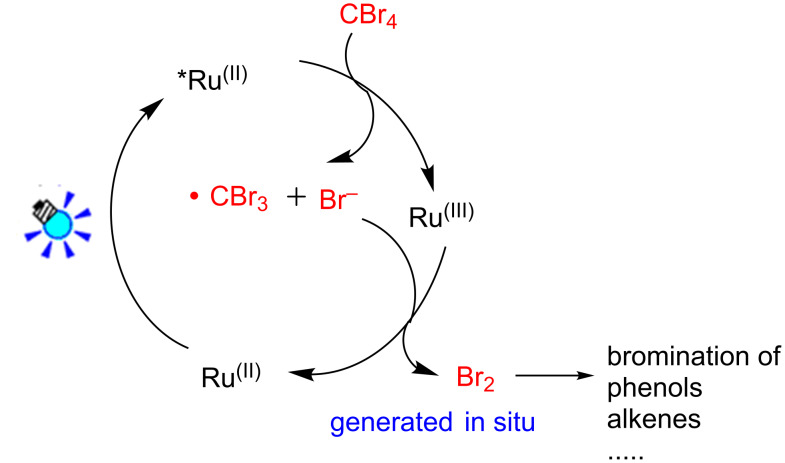
Strategy for in situ generation of bromine.

Recently, an intriguing and promising strategy for the application of photoredox catalysts to initiate single electron transfer processes have been developed [16–22]. Since the pioneering work from the groups of MacMillan [23–25], Stephenson [26–28], Yoon [29–31] and others [32–44] demonstrated the usefulness of Ru(bpy)_3_Cl_2_ and its application to various visible-light-induced synthetic transformations, visible-light-photoredox catalysis has emerged as a growing field in organic chemistry and has been successfully applied in a variety of reactions. In the oxidative quenching process [45–47], Ru(bpy)_3_^2+^ excited by visible light generates Ru(bpy)_3_^2+*^, which was oxidized to Ru(bpy)_3_^3+^ in the presence of oxidative quenchers. CBr_4_ is an example of a suitable oxidative quencher and leads to the formation of Br^−^ and •CBr_3_. We envision that Ru(bpy)_3_^3+^ is a strong oxidant (1.29 V vs SCE, in CH_3_CN) that could sequentially oxidize the resulting Br^−^ (1.087 V vs SCE, in CH_3_CN) to generate the bromine for the bromination of phenols and other substrates in situ (Scheme 1), thus avoiding the use of highly toxic and volatile liquid bromine.

## Results and Discussion

Our initial investigation was carried out on protected 4-methoxyphenol **1a** and CBr_4_ in dried CH_3_CN in the presence of Ru(bpy)_3_Cl_2_ (5.0 mol %) with visible light irradiation (blue LEDs, λ_max_ = 435 nm) for 6 hours. The corresponding 2-bromo-4-methoxyphenol (**2a**) was obtained in 78% yield (Table 1, entry 1), whereas 3-bromo-4-methoxyphenol was not observed. The optimization of the reaction conditions were conducted by screening selected solvents and the amount of the photoredox catalyst using **1a** as the representative substrate. As can be seen in Table 1, the solvent had a significant effect on the reaction efficiency. The reaction did not work well in DMF, MeOH, THF, CH_2_Cl_2_, EtOAc, CH_3_CN with 10 equivalents of H_2_O or 1,4-dioxane (Table 1, entries 5–11). The reaction in CH_3_CN and open to air led to the highest yield, 94% (Table 1, entry 4), whereas the reaction conducted under N_2_ or O_2_, or in DMSO and open to air gave lower yields (Table 1, entries 2, 3, 12). Our final optimization showed that the reaction also provided comparable results when the catalyst loading was reduced to 3% or 1% (Table 1, entry 13). It should be pointed out that an exclusion of either the photocatalyst or the light source did not afford the desired product **2a**. Therefore, the reaction conditions of CBr_4_ (1 equiv) in dried CH_3_CN in the presence of Ru(bpy)_3_Cl_2_ (5.0 mol %) with visible light irradiation (blue LEDs, λ_max_ = 435 nm) and open to air were utilized to test the scope of the reaction.

**Table 1:** Survey of the photocatalytic bromination reaction conditions.

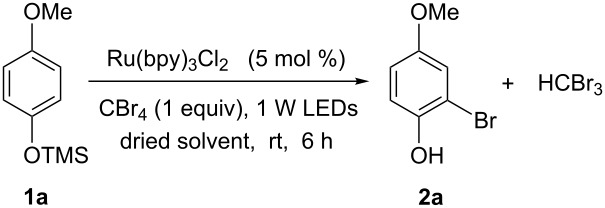		

entry	conditions^a^	yield (%)^b^

1	CH_3_CN, tube closed	78
2	CH_3_CN, N_2_	89
3	CH_3_CN, O_2_	46
**4**	**CH_3_CN, open to air**	**94**
5	CH_3_OH, open to air	23
6	CH_2_Cl_2_, open to air	0
7	DMF, open to air	63
8	THF, open to air	0
9	EtOAc, open to air	0
10	1,4-dioxane, open to air	0
11	CH_3_CN + 10 equiv H_2_O, open to air	0
12	DMSO, open to air	82
13	CH_3_CN, Ru(bpy)_3_Cl_2_ (3%), open to air	86

^a^Reaction conditions: substrate **1a** (0.1 mmol), CBr_4_ (0.1 mmol), Ru(bpy)_3_Cl_2_ (5 mol %), solvent (0.1 M), blue LEDs (1W). ^b^Yields were determined by GC analysis.

With the optimized conditions in hand, we prepared a variety of phenols which were subjected to the photocatalytic reaction. In general, both electron-withdrawing and electron–donating groups were tolerated as substituents R^2^ in this process. Interestingly, the substrates protected with TMS (trimethylsilyl), TBS (*tert*-butyldimethylsilyl), MOM (methoxymethyl) and THP (tetrahydropyranyl) groups led to the corresponding bromophenols via a Tandem bromination/deprotection reaction (Table 2, entries 1–8, 12, 13, and 15), among which the cases with substituents at *para*- and *ortho*-position afforded 2- and 4-bromophenol, respectively, in good to excellent yields (Table 2, entries 1–5 and 12). The compound substituted with a methoxy group at the *meta*-position (**1b**) led to both 2- and 4-bromophenols **2b** and **2b'** with a ratio of 2:1 (Table 2, entry 8). Without any substituent at the phenol moiety mono- and dibromophenols were obtained with a ratio of 3:2 (Table 2, entries 6 and 7). Notably, 1-bromonaphthalen-2-ol and 1-bromo-2-methoxynaphthalene could be prepared in good yields with high regioselectivity from TMS and methyl protected naphthalen-2-ol (Table 2, entries 13 and 14). The direct treatment of 3-methoxyphenol under the same reaction conditions afforded 2- and 4-bromo-3-methoxyphenol with a ratio of 3:2 in a synthetically acceptable yield (Table 2, entry 11). Phenols protected with Bn or Ms groups led to 2- and 4-bromophenol derivatives in excellent yields without the loss of Bn or Ms groups (Table 2, entries 9 and 10).

**Table 2:** Scope of the photocatalytic bromination of phenols.

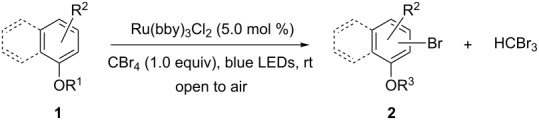			

Entry	Substrate	Product	Yield (%)^a^

	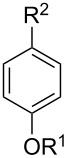	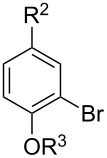	Conditions^b^
1	R^1^ = TMS; R^2^ = OMe; R^3^ = H		88
2	R^1^ = TMS; R^2^ = Me; R^3^ = H		69
3	R^1^ = TMS; R^2^ = Cl; R^3^ = H		58
4	R^1^ = TBS; R^2^ = OMe; R^3^ = H		85
5	R^1^ = MOM; R^2^ = Me; R^3^ = H		97
	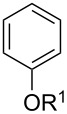	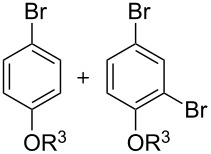	
6	R^1^ = TMS; R^3^ = H (3:2)^c^		79
7	R^1^ = THP; R^3^ = H (3:2)^c^		79
	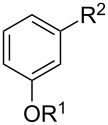	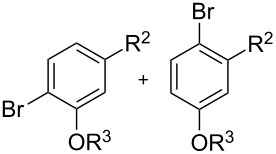	
8	R^1^ = TMS; R^2^ = OMe; R^3^ = H (2:1)^c^		73
9	R^1^ = Ms; R^2^ = OMe; R^3^ = Ms (5:1)^c^		95
10	R^1^ = Bn; R^2^ = OMe; R^3^ = Bn (5:3)^c^		98
11	R^1^ = H; R^2^ = OMe; R^3^ = H (3:2)^c^		40
12	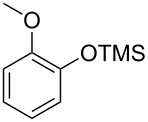	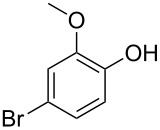	84
	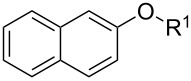	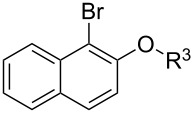	
13	R^1^ = TMS; R^3^ = H		76
14	R^1^ = Me; R^3^ = Me		98
15	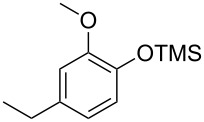	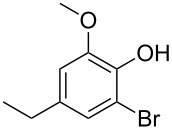	46

^a^Isolated yield based on complete consume of the starting material. ^b^Reaction conditions: substrate **1** (0.1 mmol), CBr_4_ (0.1 mmol), Ru(bpy)_3_Cl_2_ (5 mol %), dried CH_3_CN, blue LEDs (1 W), open to air. ^c^Ratio of the isomers in parentheses.

**Scheme 2:**
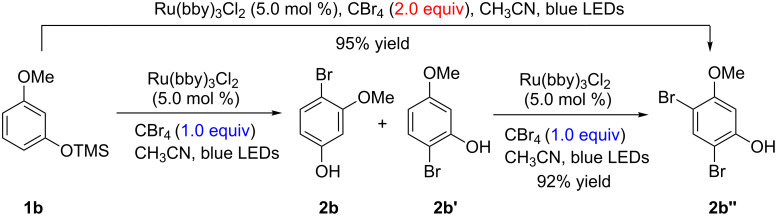
Synthesis of dibromophenol product **2b''**.

The bromination of phenols could be controlled by the amount of CBr_4_. For example, when TMS protected 3-methoxyphenol was treated with 2 equivalents of CBr_4_ under similar conditions (Table 2), a dibromophenol product was directly obtained in a high yield (95%) (Scheme 2), which also could be prepared from the same starting materials in two steps (Table 2).

**Scheme 3:**
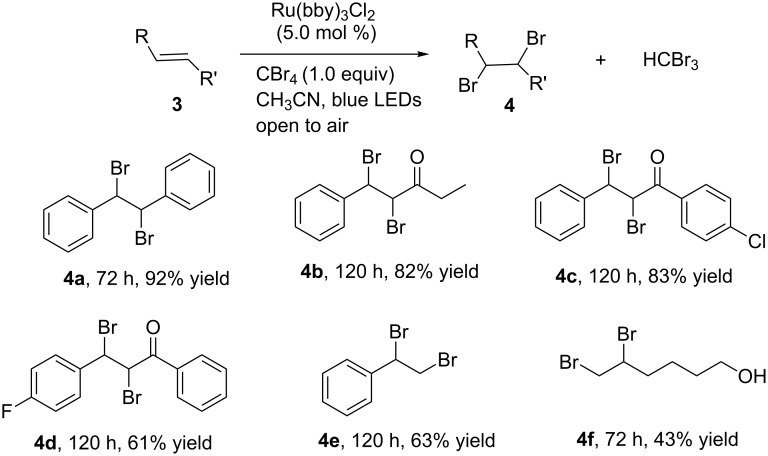
Scope of the photocatalytic bromination of alkenes.

We also conducted a control experiment by reacting stilbene with CBr_4_ (1 equiv) in dry CH_3_CN in the presence of Ru(bpy)_3_Cl_2_ (5.0 mol %) with visible-light irradiation (blue LEDs, λ_max_ = 435 nm) for 72 hours, which led to the *anti*-1,2-dibromo-1,2-diphenylethane in 92% yield. This result is in accordance with the direct bromination of stilbene from liquid bromine [47]. Based on this result, our protocol provides an easily manageable and environment-friendly pathway to the bromination of alkenes. We further examined the scope of the reaction, and the results are summarized in Scheme 3. The 1,2-dibromo products were obtained in moderate to high yields.

**Scheme 4:**
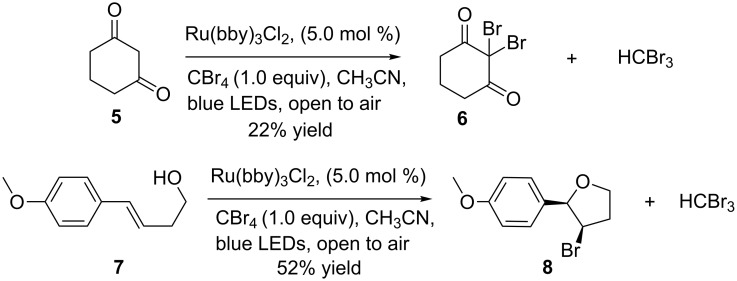
Bromination of diketones and cyclization reactions.

With the success of the bromination of phenols and alkenes, we further focused on the complementary bromination of diketones and cyclization reactions. The treatment of cyclohexane-1,3-dione (**5**) and (*E*)-4-(4-methoxyphenyl)but-3-en-1-ol (**7**) under the same reaction conditions led to 2,2-dibromocyclohexane-1,3-dione (**6**) and bromofuran compound **8** in 22% and 52% yield, respectively (Scheme 4). This outcome demonstrates the efficiency of the Ru(bpy)_3_Cl_2_/CBr_4_ photocatalytic system. The stereochemistry of the bromofuran compound was determined by 2D NMR spectra.

## Conclusion

In summary, we have developed a mild and operationally simple method for the in situ preparation of bromine utilizing a visible-light photoredox catalyst. The reaction proceeds with high chemical yield and regioselectivity for the bromination of phenols and alkenes. Further development of photoredox catalysis in the context of radical chemistry and its application in other reactions are currently underway in our laboratory.

## Experimental

### General procedure for the bromination of phenols and alkenes


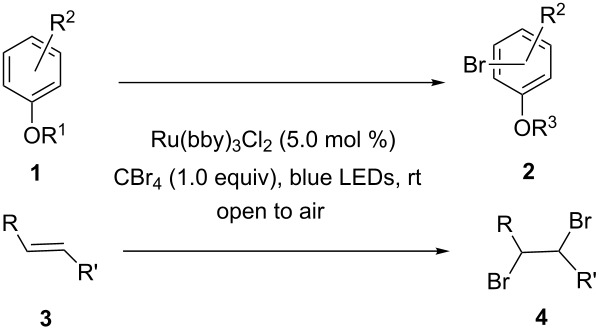


To a 10 mL round bottom flask equipped with a magnetic stir bar were added phenols or alkenes (0.1 mmol), CBr_4_ (33 mg, 0.1 mmol), dry CH_3_CN (1 mL) and Ru(bpy)_3_Cl_2_ (3.8 mg, 0.005 mmol). The mixture was irradiated with blue LEDs (1 W) at room temperature open to air until the starting material disappeared completely (monitored by TLC). After the reaction was completed, the solvent was concentrated in vacuo. The residue was purified by flash column chromatography to give the final product.

### Bromination of diketones


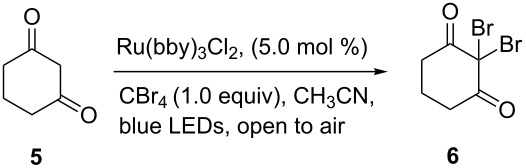


To a 10 mL round bottom flask equipped with a magnetic stir bar were added **5** (0.4 mmol), CBr_4_ (133 mg, 0.4 mmol), dry CH_3_CN (2 mL) and Ru(bpy)_3_Cl_2_ (15 mg, 0.02 mmol). The mixture was irradiated with blue LEDs (1 W) at room temperature open to air until the starting material was largely consumed (monitored by TLC). After the reaction was completed the solvent was concentrated in vacuo. The residue was purified by flash column chromatography to give the final product **6**.

### Synthesis of bromofuran


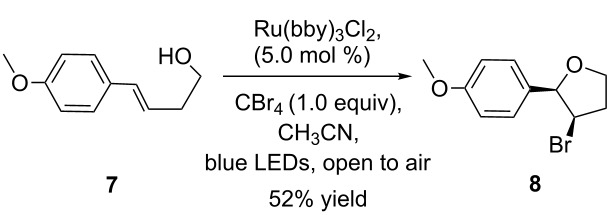


To a 10 mL round bottom flask equipped with a magnetic stir bar were added **7** (0.13 mmol), CBr_4_ (43 mg, 0.13 mmol), LiBr (11 mg, 0.13 mmol), dry CH_3_CN (1 mL) and Ru(bpy)_3_Cl_2_ (4.5 mg, 0.006 mmol). The mixture was irradiated with blue LEDs (1 W) at room temperature open to air until the starting material disappeared completely (monitored by TLC). After the reaction was completed the solvent was concentrated in vacuo. The residue was purified by flash column chromatography to give the final product **8**.

## Supporting Information

File 1 ^1^H and ^13^C NMR spectra for products.
