# Supplementary material for: Visible-light photoredox catalysis enabled bromination of phenols and alkenes
Source: Beilstein J Org Chem. 2014 Mar 7;10:622–7. doi: 10.3762/bjoc.10.53 (PMC3999835; doi:10.3762/bjoc.10.53)
Supplement: File 1 — 1H and 13C NMR spectra for products. [file Beilstein_J_Org_Chem-10-622-s001.pdf]

Supporting Information

for

Visible-light photoredox catalysis enabled bromination of phenols and alkenes

Yating Zhao, Zhe Li, Chao Yang, Run Lin, and Wujiong Xia*

Address: State Key Lab of Urban Water Resource and Environment, The Academy of Fundamental and Interdisciplinary Sciences, Harbin Institute of Technology, Harbin 150080, China

Email: Wujiong Xia - xiawj@hit.edu.cn

* Corresponding author

^{1}H and ^{13}C NMR spectra for products.

General Information

All reagents were purchased from commercial sources unless otherwise noted. Solvents were dried according to standard procedures prior to use. ^{1}H NMR (400 MHz) and ^{13}C NMR (100 MHz) spectra were recorded on a Bruker AV-400 spectrometer in $CDCl_3$. For ^{1}H NMR (400 MHz), tetramethylsilane (TMS) served as an internal standard (δ = 0 ppm), and data are reported as follows: chemical shift (in ppm), multiplicity (s = singlet, d = doublet, t = triplet, q = quartet, m = multiplet, br =

broad), coupling constant (in Hz), and integration. For ^{13}C NMR (100 MHz), $CDCl_3$ was used as an internal standard (δ = 77.23 ppm) and spectra were obtained with complete proton decoupling. HRMS spectra were recorded on a Bruker Esquire LC mass spectrometer by using electrospray ionization (ESI, TOF).

Characterization data of all products

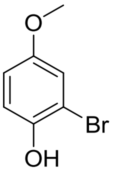

2-Bromo-4-methoxyphenol (2a): ^1H NMR (400 MHz, $CDCl_3$): *δ* 7.01 (d, *J* = 2.88 Hz, 1H), 6.94 (d, *J* = 8.91 Hz, 1H), 6.78 (dd, *J* = 2.91, 2.88 Hz, 1H), 5.23 (s, 1H), 3.75 (s, 3H). ^{13}C NMR (100 MHz, $CDCl_3$): *δ* 153.83, 146.53, 116.85, 116.33, 115.35, 109.92, 55.99.

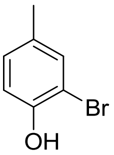

2-Bromo-4-methylphenol (2c): ^1H NMR (400 MHz, $CDCl_3$): *δ* 7.27 (s, 1H), 7.01 (d, *J* = 8.25 Hz, 1H), 6.90 (d, *J* = 8.24 Hz, 1H), 5.37 (s, 1H), 2.27 (s, 1H). ^{13}C NMR (100 MHz, $CDCl_3$): *δ* 150.03, 132.12, 131.41, 129.77, 115.74, 109.83, 20.19.

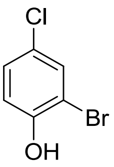

2-Bromo-4-chlorophenol (2d): ^1H NMR (400 MHz, $CDCl_3$): *δ* 7.46 (d, *J* =2.06 Hz, 1H), 7.19 (dd, *J* = 2.44, 2.43 Hz, 1H), 6.95 (d, *J* = 8.71 Hz, 1H), 5.58 (s, 1H). ^{13}C NMR (100 MHz, $CDCl_3$): *δ* 151.20, 131.59, 131.35, 129.23, 116.89, 116.05.

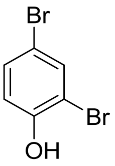

2,4-Bibromophenol (2e): ^1H NMR (400 MHz, $CDCl_3$): *δ* 7.59 (d, *J* =2.25 Hz, 1H), 7.32 (dd, *J* =2.25, 2.24 Hz, 1H), 6.91 (d, *J* = 8.69 Hz, 1H), 5.89 (s, 1H). ^{13}C NMR (100 MHz, $CDCl_3$): *δ* 151.83, 134.12, 132.06, 117.48, 112.52, 110.87.

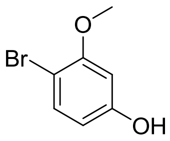
4-Bromo-3-methoxyphenol (2b): ^{1}H NMR (400 MHz, $CDCl_3$): δ 7.34 (d, J = 8.52 Hz, 1H), 6.60 (d, J = 2.85 Hz, 1H), 6.42 (dd, J = 2.88, 2.85 Hz, 1H), 5.49 (s, 1H), 3.77 (s, 3H). ^{13}C NMR (100 MHz, $CDCl_3$): δ 156.76, 156.13, 133.36, 108.51, 102.27, 100.46, 56.19.

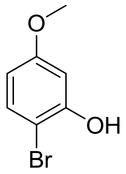
2-Bromo-5-methoxyphenol (2b'): ^{1}H NMR (400 MHz, $CDCl_3$): δ 7.32 (d, J =8.85 Hz, 1H), 6.45 (d, J =2.68 Hz, 1H), 6.34 (dd, J =2.71, 2.71 Hz, 1H), 4.94 (s, 1H), 3.86 (s, 3H). ^{13}C NMR (100 MHz, $CDCl_3$): δ 160.61, 153.00, 131.95, 108.45, 101.67, 100.89, 55.55.

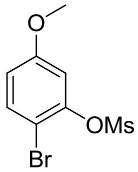
2-Bromo-5-methoxyphenyl methanesulfonate (2f): ^{1}H NMR (400 MHz, $CDCl_3$): δ 7.49 (d, J =8.12 Hz, 1H), 7.02 (d, J =2.82 Hz, 1H), 6.77 (dd, J =2.84, 2.84 Hz, 1H), 3.81 (s, 3H), 3.26 (s, 3H). ^{13}C NMR (100 MHz, $CDCl_3$): δ 159.90, 146.95, 133.75, 114.98, 110.08, 105.82, 55.87, 38.86.

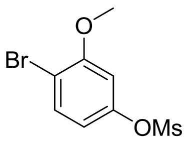
4-Bromo-3-methoxyphenyl methanesulfonate (2g): ^{1}H NMR (400 MHz, $CDCl_3$): δ 7.49 (d, J =8.92 Hz, 1H), 7.01 (d, J =2.82 Hz, 1H), 6.77 (dd, J = 2.84, 2.85 Hz, 1H), 3.81 (s, 3H), 3.26 (s, 3H). ^{13}C NMR (100 MHz, $CDCl_3$): δ 159.90, 146.94, 133.74, 114.97, 110.08, 105.82, 55.87, 38.86.

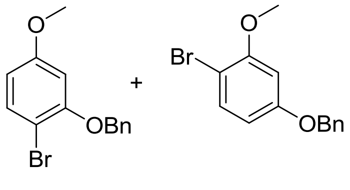
2-(Benzyloxy)-1-bromo-4-methoxybenzene (2h) and 4-(benzyloxy)-1-bromo-2-methoxy-benzene (2i): ^{1}H NMR (400 MHz, $CDCl_3$): δ 7.49-7.33 (m, 10H), 6.58 (d, J =2.62 Hz, 1H), 6.54 (d, J =2.66 Hz, 0.65H), 6.47 (dd, J = 2.68, 2.67Hz, 1H), 6.42 (dd, J = 2.69, 2.70 Hz, 0.65H), 5.13 (s, 1.30 H), 5.05 (s, 2H), 3.85 (s, 3H), 3.76 (s, 2.56H). ^{13}C NMR (100 MHz, $CDCl_3$): δ 160.10, 159.41,

156.62, 155.71, 136.57, 136.46, 133.24, 133.19, 128.69, 128.62, 128.19, 127.98, 127.56, 127.06, 106.90, 106.57, 103.29, 102.78, 101.73, 100.94, 70.81, 70.42, 56.17, 55.56.

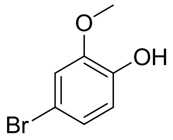

4-Bromo-2-methoxyphenol (2j): ^{1}H NMR (400 MHz, $CDCl_3$): δ 7.00-6.97 (m, 2H), 6.80 (d, *J* =8.24 Hz, 1H), 5.51 (s, 1H), 3.88 (s, 3H). ^{13}C NMR (100 MHz, $CDCl_3$): δ 147.23, 144.91, 124.19, 115.75, 114.18, 111.57, 56.16.

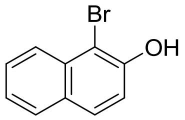

1-Bromonaphthalen-2-ol (2k): ^{1}H NMR (400 MHz, $CDCl_3$): δ 8.02 (d, *J* =8.48 Hz, 1H), 7.77 (d, *J* =8.16 Hz, 1H), 7.73 (d, *J* =8.45 Hz, 1H), 7.56 (t, *J* =15.40 Hz, 1H), 7.38 (t, *J* =15.03 Hz, 1H), 7.26 (d, *J* =8.85 Hz, 1H), 5.93 (s, 1H). ^{13}C NMR (100 MHz, $CDCl_3$): δ 150.61, 132.32, 129.71, 129.35, 128.23, 127.86, 125.35, 124.16, 117.18, 106.16.

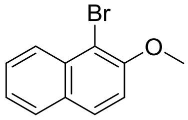

1-Bromo-2-methoxynaphthalene (2l): ^{1}H NMR (400 MHz, $CDCl_3$): δ 8.23 (d, *J* = 8.60 Hz, 1H), 7.84-7.73 (m, 3H), 7.597 (t, *J* = 15.42 Hz, 1H), 7.40 (t, *J* = 15.05 Hz, 1H), 7.28 (d, *J* = 8.99 Hz, 1H), 4.04 (s, 1H). ^{13}C NMR (100 MHz, $CDCl_3$): δ 153.79, 133.16, 129.86, 128.97, 128.05, 127.75, 126.16, 124.34, 113.68, 108.73, 57.09.

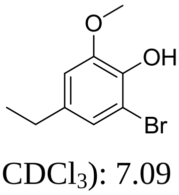

2-Bromo-4-ethyl-6-methoxyphenol (2m): ^{1}H NMR (400 MHz, $CDCl_3$): 7.09 (s, 1H), 6.71 (s, 1H), 5.48 (s, 1H), 3.87 (s, 3H), 2.70-2.64 (m, 2H), 1.21-1.17 (t, *J* =15.04 Hz, 3H). ^{13}C NMR (100 MHz, $CDCl_3$): δ 146.03, 144.27, 134.72, 118.43, 114.36, 111.47, 56.07, 29.11, 14.68.

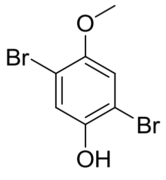

2,5-Dibromo-4-methoxyphenol (2n): ^{1}H NMR (400 MHz, $CDCl_3$): δ 7.12 (d, *J* =2.95 Hz, 1H), 6.80 (d, *J* =2.96 Hz, 1H), 5.65 (s, 1H), 3.78 (s, 3H). ^{13}C NMR (100 MHz, $CDCl_3$): δ 153.73, 143.51, 126.08, 117.76, 116.61, 111.54, 56.02.

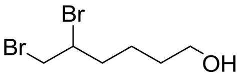 **5,6-Dibromohexan-1-ol** [1] **(4f):** ^{1}H NMR (400 MHz, $CDCl_3$) : δ 4.22-4.13 (m, 1H), 3.85 (dd, J = 10.3, 4.4 Hz, 1H), 3.65 (dt, J = 20.3, 6.3 Hz, 3H), 2.24-2.10 (m, 1H), 1.89-1.67 (m, 2H), 1.65-1.48 (m, 4H). ^{13}C NMR (100 MHz, $CDCl_3$) : δ 62.71 (s), 52.97 (s), 36.39 (s), 36.00 (s), 32.03 (s), 23.36 (s).

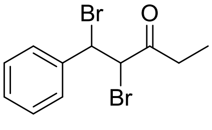 **1,2-Dibromo-1-phenylpentan-3-one (4b):** ^{1}H NMR (400 MHz, $CDCl_3$) : δ 7.44-7.36 (m, 5H), 5.37 (d, J = 11.6 Hz, 1H), 4.95 (d, J = 11.6 Hz, 1H), 2.87 (q, J = 7.2 Hz, 1H), 2.76 (q, J = 7.2 Hz, 1H), 1.23 (t, J = 7.2 Hz, 3H). ^{13}C NMR (100 MHz, $CDCl_3$): δ 201.79 (s), 138.09 (s), 129.45 (s), 129.01 (s), 128.29 (s), 51.83 (s), 49.71 (s), 34.00 (s), 8.03 (s).

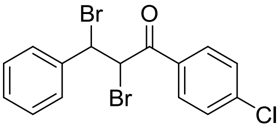

2,3-Dibromo-1-(4-chlorophenyl)-3-phenylpropan-1-one [2] **(4c):** ^{1}H NMR (400 MHz, Acetone): δ 8.35-8.25 (m, 2H), 7.79-7.73 (m, 2H), 7.70-7.64 (m, 2H), 7.44 (dt, J = 19.5, 7.0 Hz, 3H), 6.49 (d, J = 11.3 Hz, 1H), 5.74 (d, J = 11.3 Hz, 1H). ^{13}C NMR (100 MHz, Acetone) : δ 206.32 (s), 131.65 (s), 130.15 (d, J = 14.1 Hz), 129.59 (d, J = 6.3 Hz), 51.16 (s), 47.26 (s), 29.84 (s).

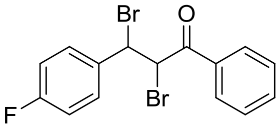

2,3-Dibromo-3-(4-fluorophenyl)-1-phenylpropan-1-one (4d): ^{1}H NMR (400 MHz, $CDCl_3$): δ 8.03 (d, J = 8.7 Hz, 2H), 7.57-7.46 (m, 4H), 7.12 (t, J = 8.6 Hz, 2H), 5.70 (d, J = 11.3 Hz, 1H), 5.61 (d, J = 11.3 Hz, 1H). ^{13}C NMR (100 MHz, $CDCl_3$): δ 190.03 (s), 164.34 (s), 141.08 (s), 134.23 (s), 132.80 (s), 130.38 (d, J = 10.4 Hz), 129.58 (s), 116.02 (s), 48.91 (s), 47.03 (s).

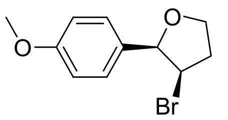

3-Bromo-2-(4-methoxyphenyl)tetrahydrofuran (8): ^{1}H NMR (400 MHz, $CDCl_3$) : δ 7.30 (d, J = 8.5 Hz, 2H), 6.89 (d, J = 8.8 Hz, 2H), 5.05 (d, J = 5.0 Hz, 1H), 4.23 (td, J = 8.2, 4.2 Hz, 1H), 4.14 (ddd, J = 9.9, 9.1, 6.4 Hz, 2H), 3.80 (s, 3H), 2.62-2.51 (m, 1H), 2.31 (ddt, J = 13.6, 7.1, 4.4 Hz, 1H). ^{13}C NMR (100 MHz, $CDCl_3$) : δ 159.61 (s), 132.00 (s), 127.15 (s), 114.06 (s), 88.35 (s), 67.47 (s), 55.43 (s), 52.87 (s), 36.38 (s).

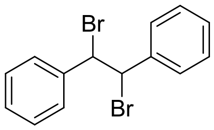

1,2-Dibromo-1,2-diphenylethane [3] **(4a):** ^{1}H NMR (400 MHz, $CDCl_3$): δ 7.18 (s, 5H), 5.48 (s, 1H). ^{13}C NMR (100 MHz, $CDCl_3$): δ 137.92 (s), 128.72 (d, J = 2.9 Hz), 128.31 (s), 59.25 (s).

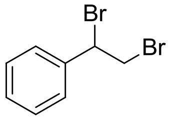

1,2-Dibromo-1-phenylethane [4] **(4e):** ^{1}H NMR (400 MHz, $CDCl_3$): δ 7.44-7.34 (m, 5H), 5.15 (dd, J = 10.6, 5.5 Hz, 1H), 4.13-3.99 (m, 2H). ^{13}C NMR (100 MHz, $CDCl_3$): δ 138.64 (s), 129.21 (s), 128.89 (s), 127.68 (s), 50.89 (s), 35.04 (s).

References:

1. G. I. Nikishin, L. L. Sokova, N. I. Kapustina, *Russian Chemical Bulletin*, **2012**, *61*, 459.
2. M. A. Rekhter, G. N. Grushetskaya, A. A. Panasenko, M. Z. Krimer, *Chemistry of Heterocyclic Compounds*, **1995**, *31*, 792.
3. Kefeng Ma, Shaw Li, Richard G. Weiss, *Org. Lett.*, **2008**, *10*, 4155.
4. Gajanan K. Dewkar, Srinivasarao V. Narina, Arumugam Sudalai, *Org. Lett.*, **2003**, *5*, 4501.

^{1}H NMR and ^{13}C NMR spectra for bromophenols and other bromoproducts

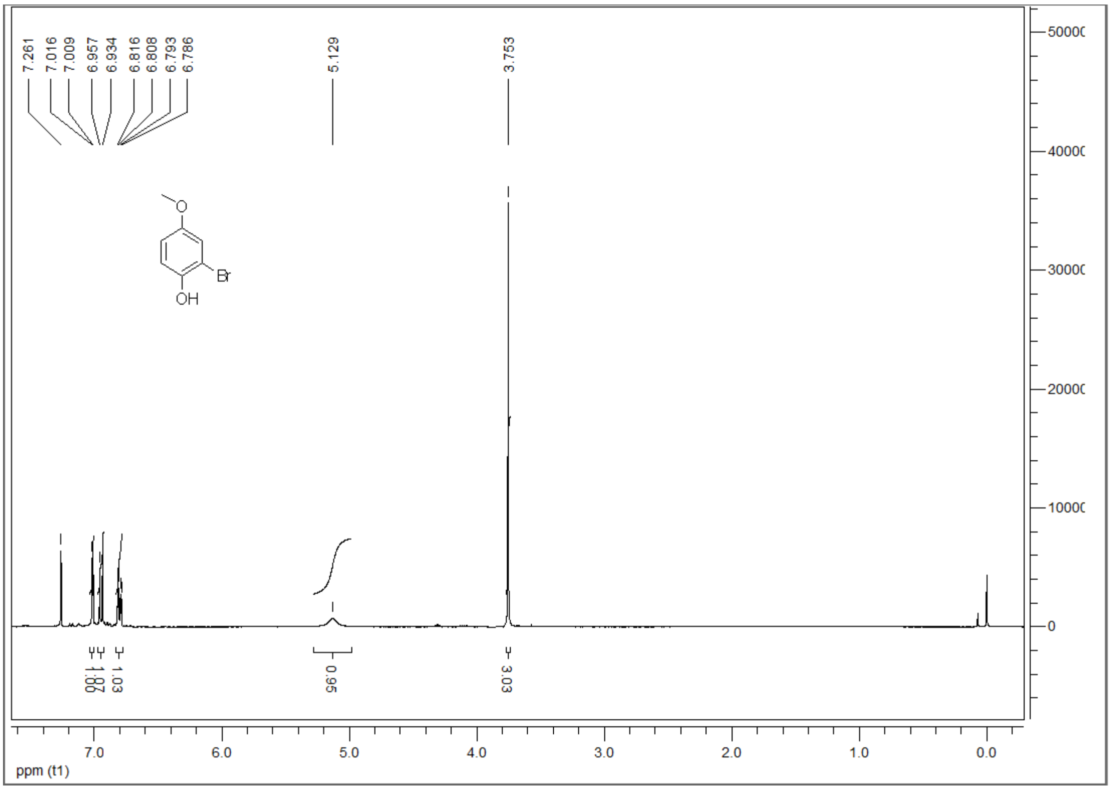

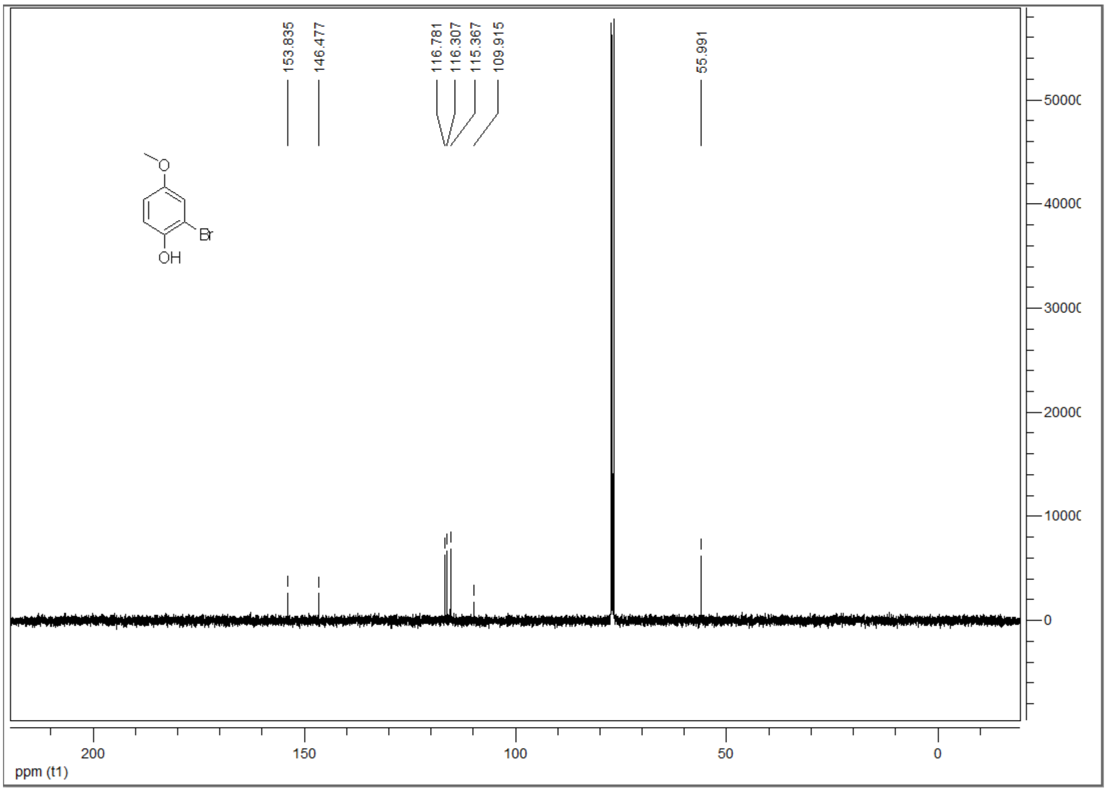

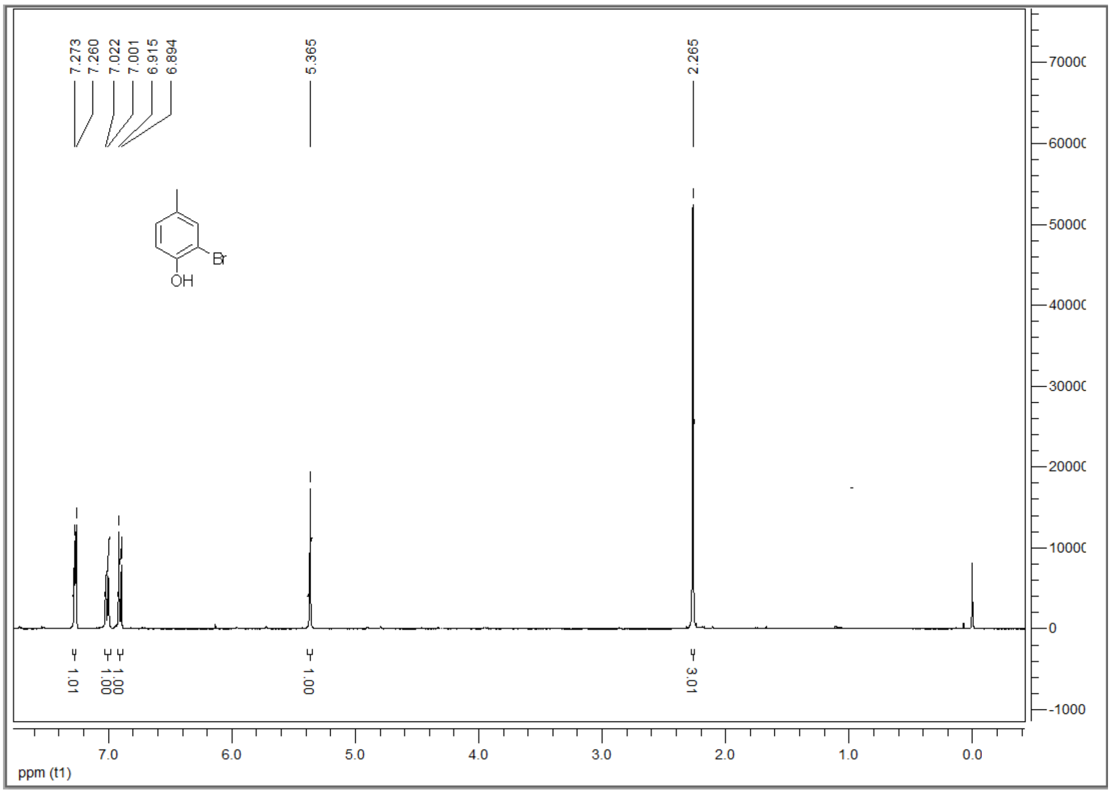
7.273
7.260
7.022
7.001
6.915
6.894
5.365
2.265
Br
OH
1.01
1.00
1.00
1.00
3.01
ppm (t1)

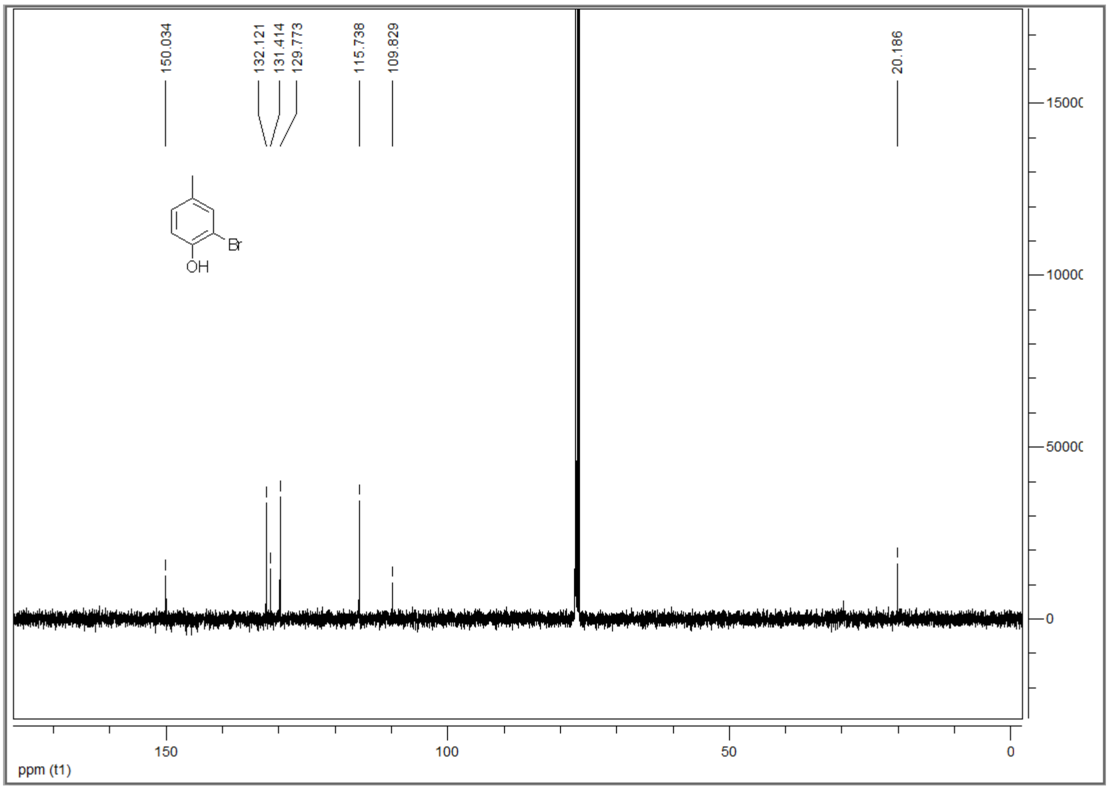
150.034
132.121
131.414
129.773
115.738
109.829
20.186
Br
OH
ppm (t1)

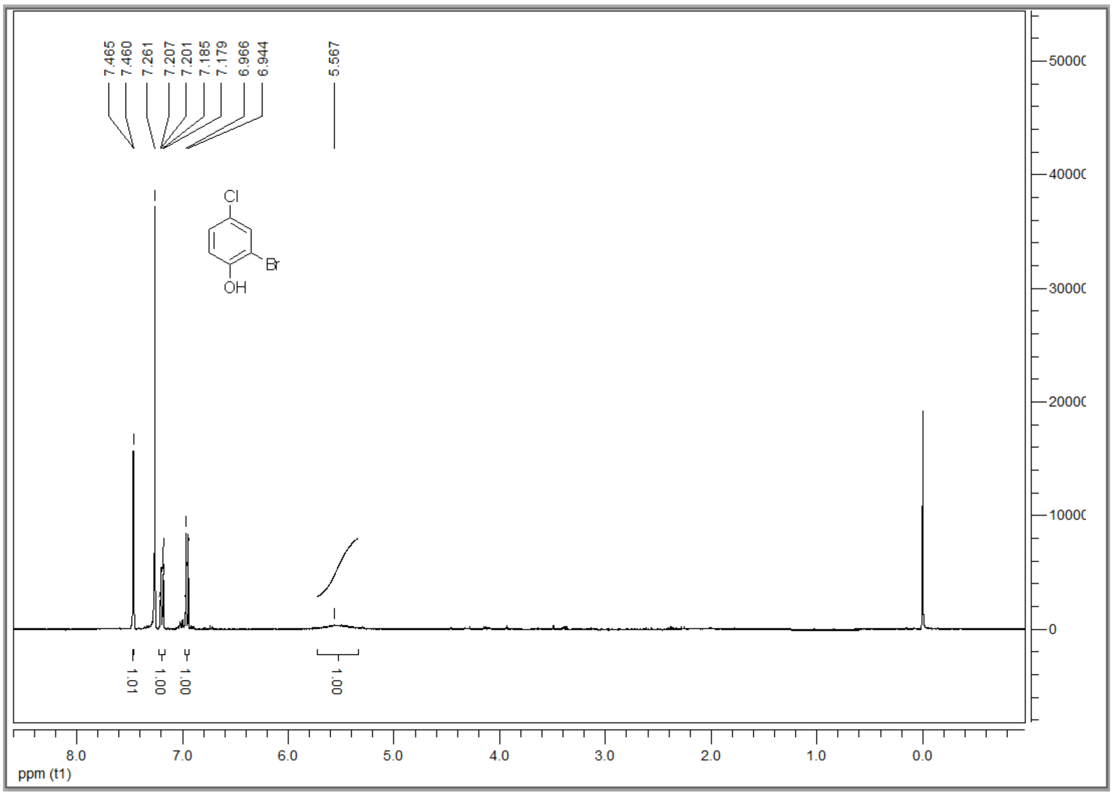
7.465
7.460
7.261
7.207
7.201
7.185
7.179
6.966
6.944
5.567
Cl
Br
OH
1.01
1.00
1.00
1.00
50000
40000
30000
20000
10000
0
8.0
7.0
6.0
5.0
4.0
3.0
2.0
1.0
0.0
ppm (t1)

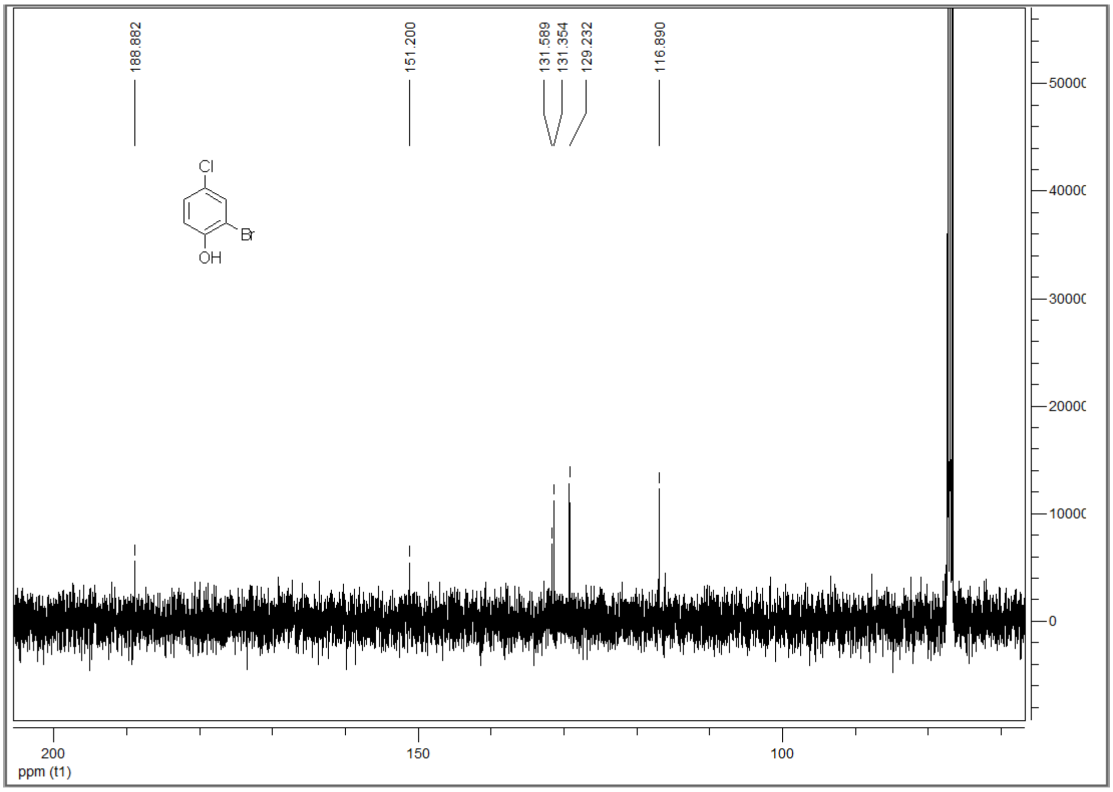
188.882
151.200
131.589
131.354
129.232
116.890
Cl
Br
OH
50000
40000
30000
20000
10000
0
200
150
100
ppm (t1)

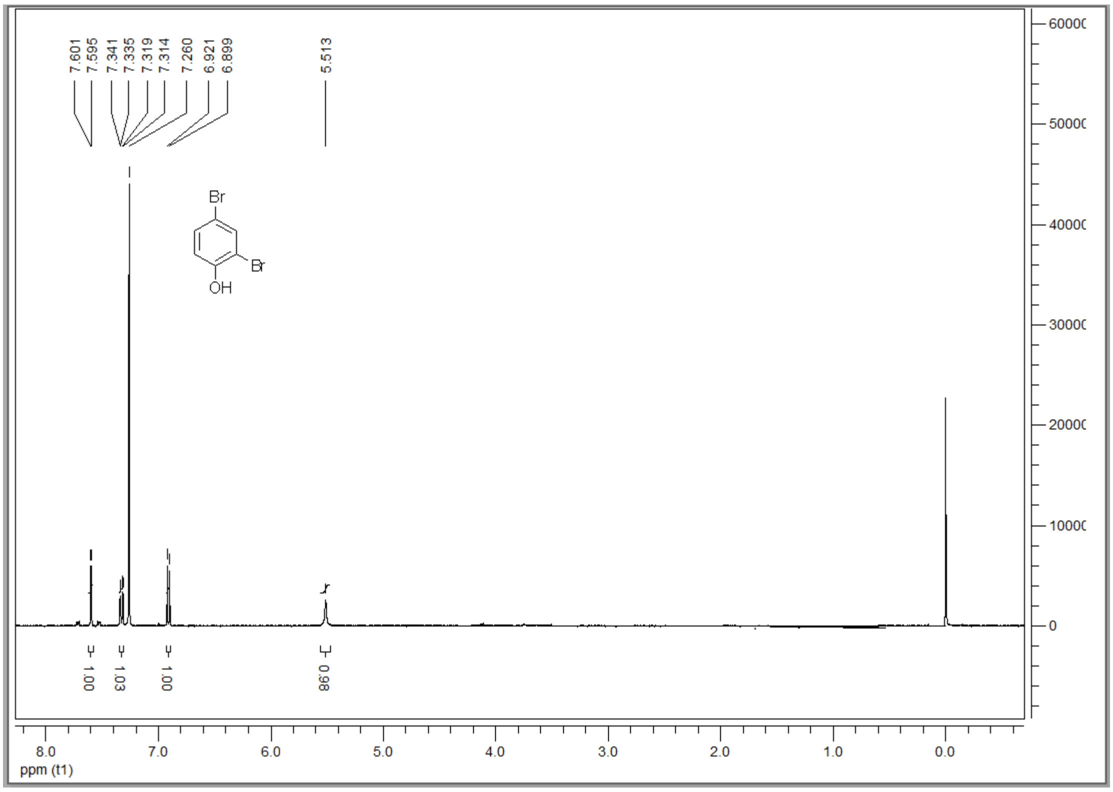
7.601
7.595
7.341
7.335
7.319
7.314
7.260
6.921
6.899
5.513
Br
Br
OH
1.00
1.03
1.00
0.98
8.0
7.0
6.0
5.0
4.0
3.0
2.0
1.0
0.0
ppm (t1)

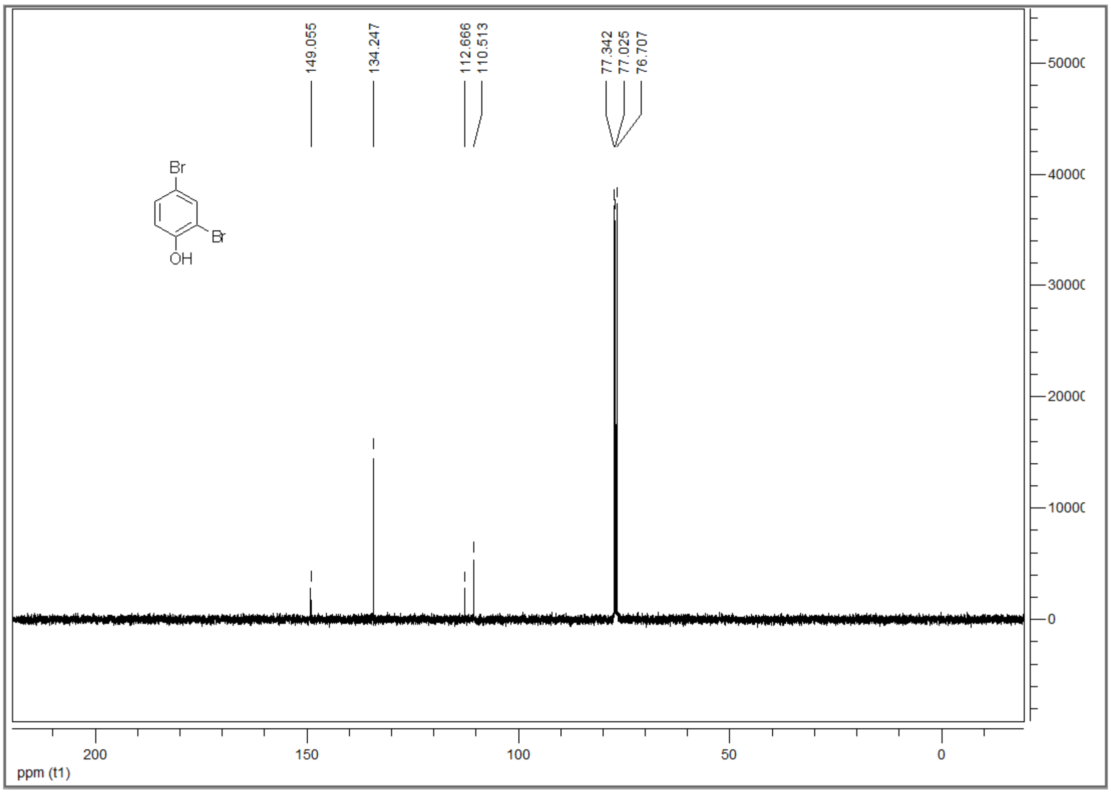
149.055
134.247
112.666
110.513
77.342
77.025
76.707
Br
Br
OH
200
150
100
50
0
ppm (t1)

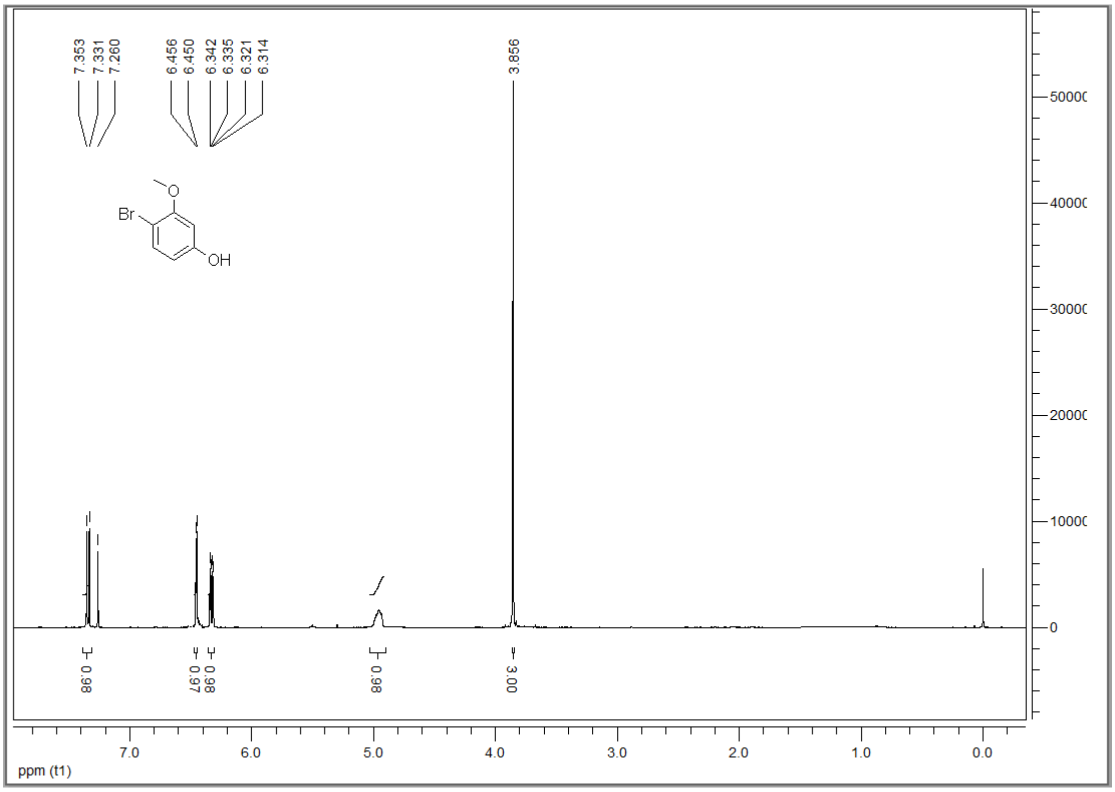
7.353
7.331
7.260
6.456
6.450
6.342
6.335
6.321
6.314
3.856
Br
O
OH
0.98
0.97
0.98
0.98
3.00
7.0
6.0
5.0
4.0
3.0
2.0
1.0
0.0
ppm (t1)

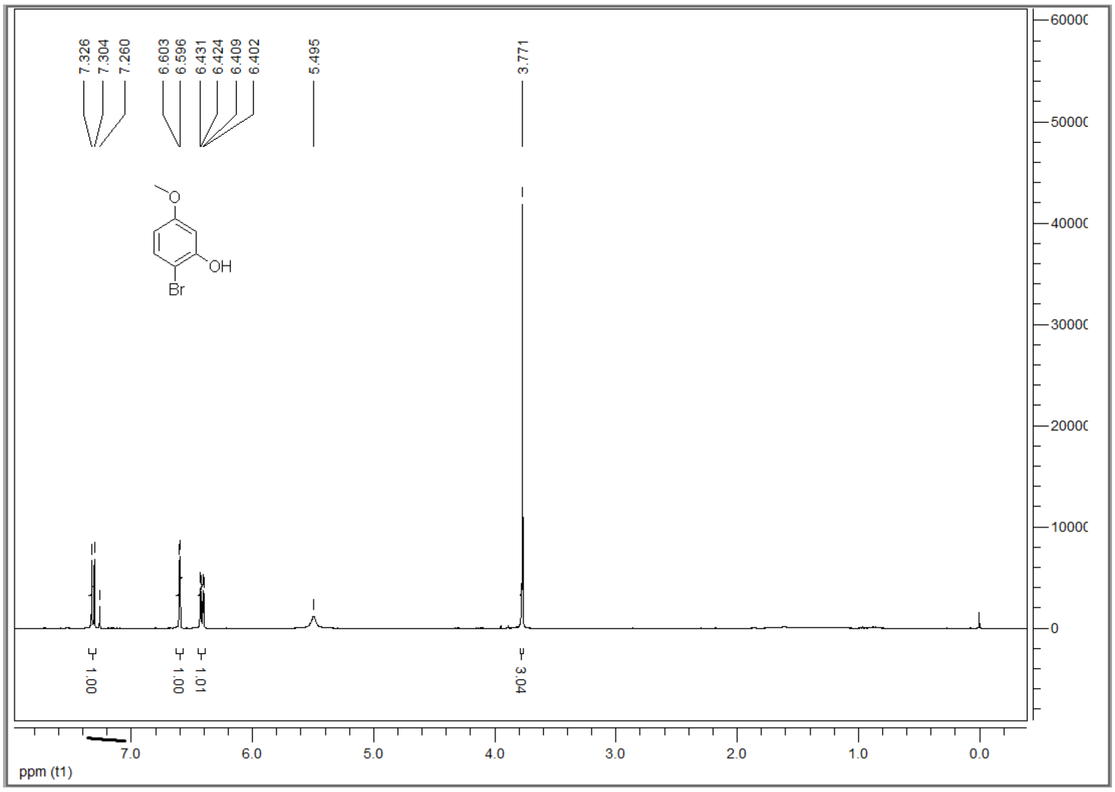
7.326
7.304
7.260
6.603
6.596
6.431
6.424
6.409
6.402
5.495
3.771
OH
Br
1.00
1.00
1.01
3.04
7.0
6.0
5.0
4.0
3.0
2.0
1.0
0.0
ppm (t1)

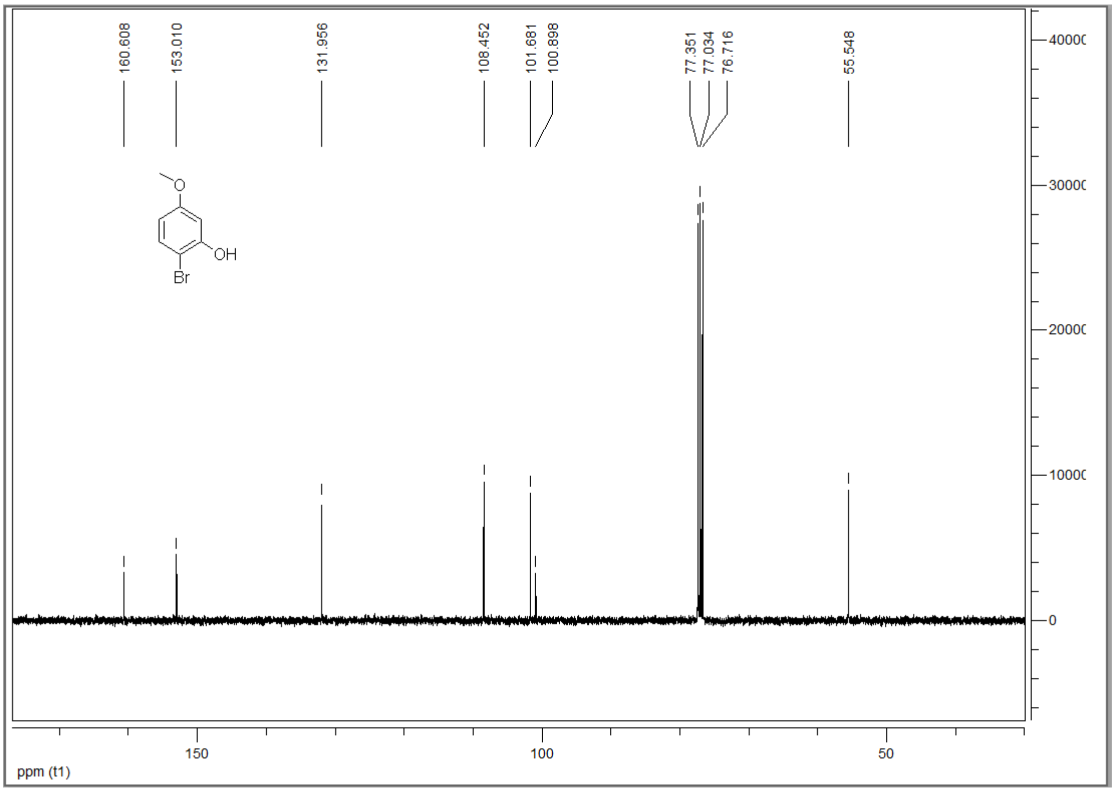
160.608
153.010
131.956
108.452
101.681
100.898
77.351
77.034
76.716
55.548
OH
Br
150
100
50
ppm (t1)

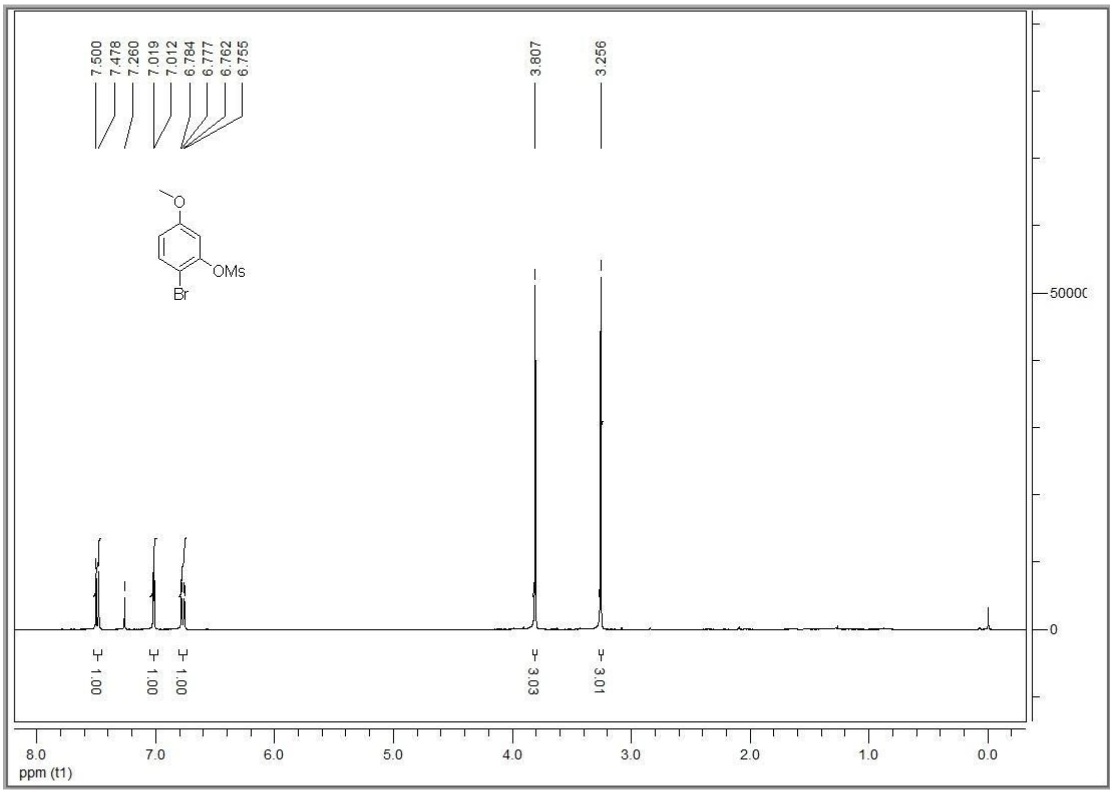
7.500
7.478
7.260
7.019
7.012
6.784
6.777
6.762
6.755
3.807
3.256
OMs
Br
50000
0
1.00
1.00
1.00
3.03
3.01
8.0
7.0
6.0
5.0
4.0
3.0
2.0
1.0
0.0
ppm (t1)

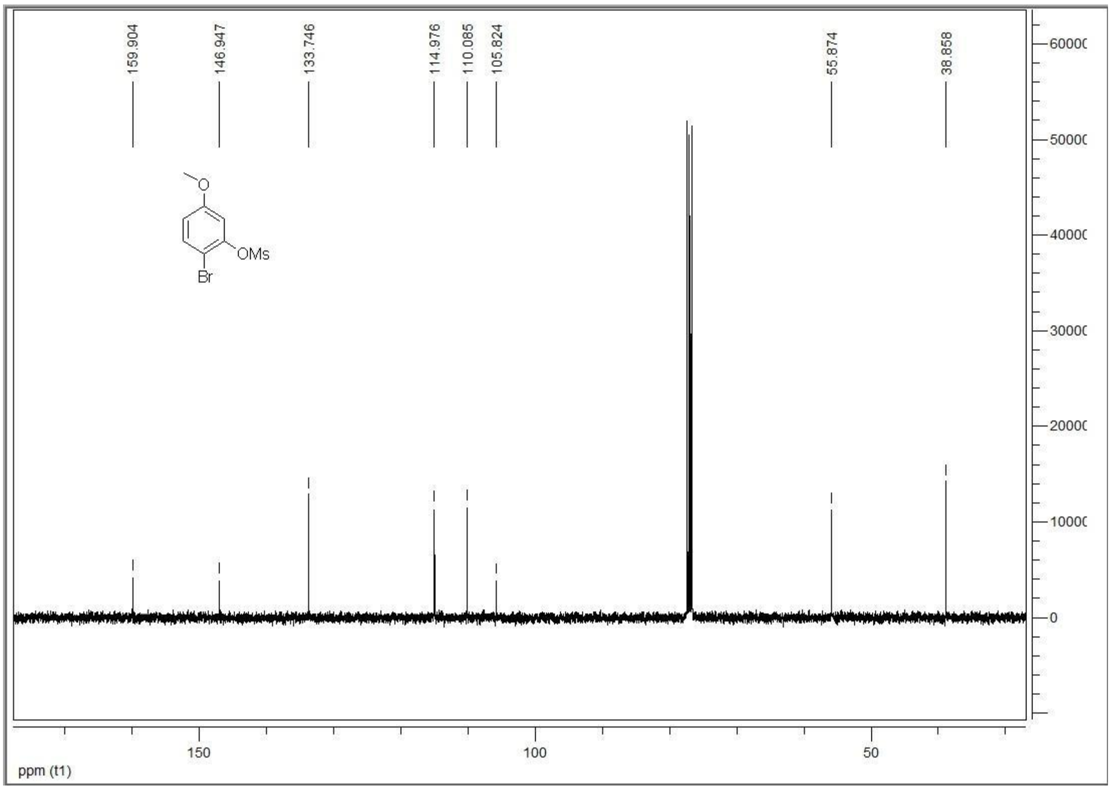
159.904
146.947
133.746
114.976
110.085
105.824
55.874
38.858
OMs
Br
60000
50000
40000
30000
20000
10000
0
150
100
50
ppm (t1)

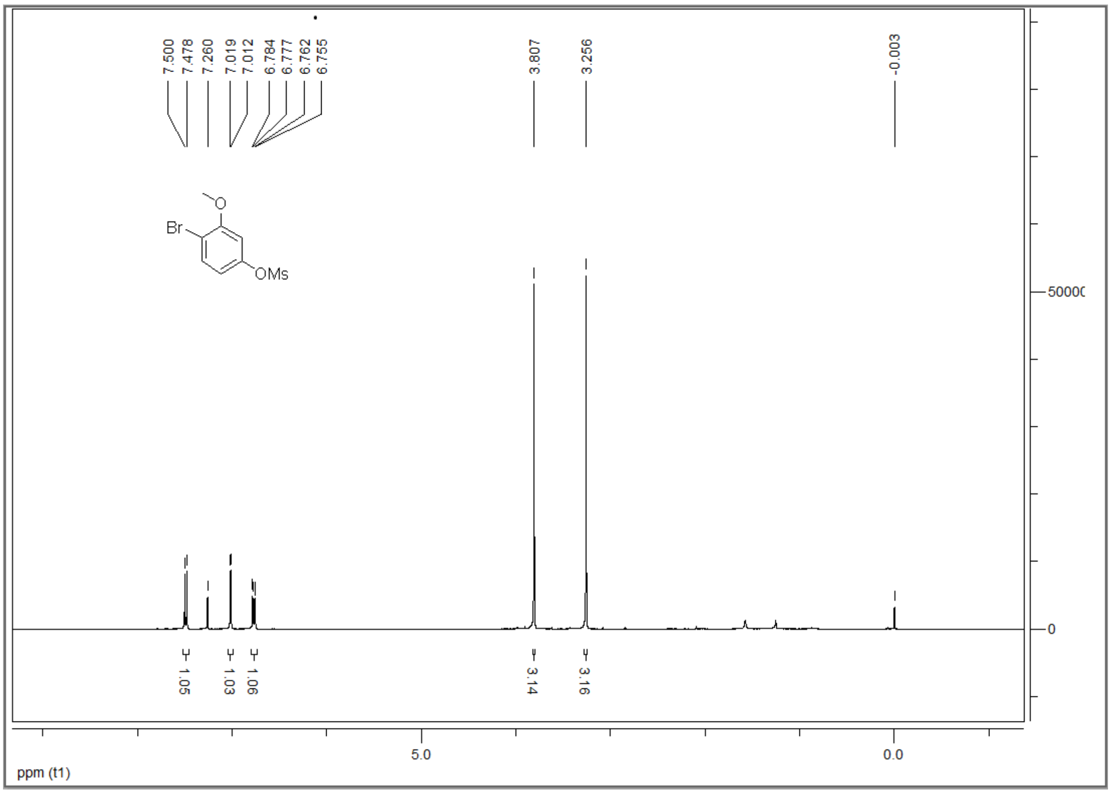

7.500
7.478
7.260
7.019
7.012
6.784
6.777
6.762
6.755
3.807
3.256
-0.003
Br
O
OMs
50000
0
1.05
1.03
1.06
3.14
3.16
5.0
0.0
ppm (t1)

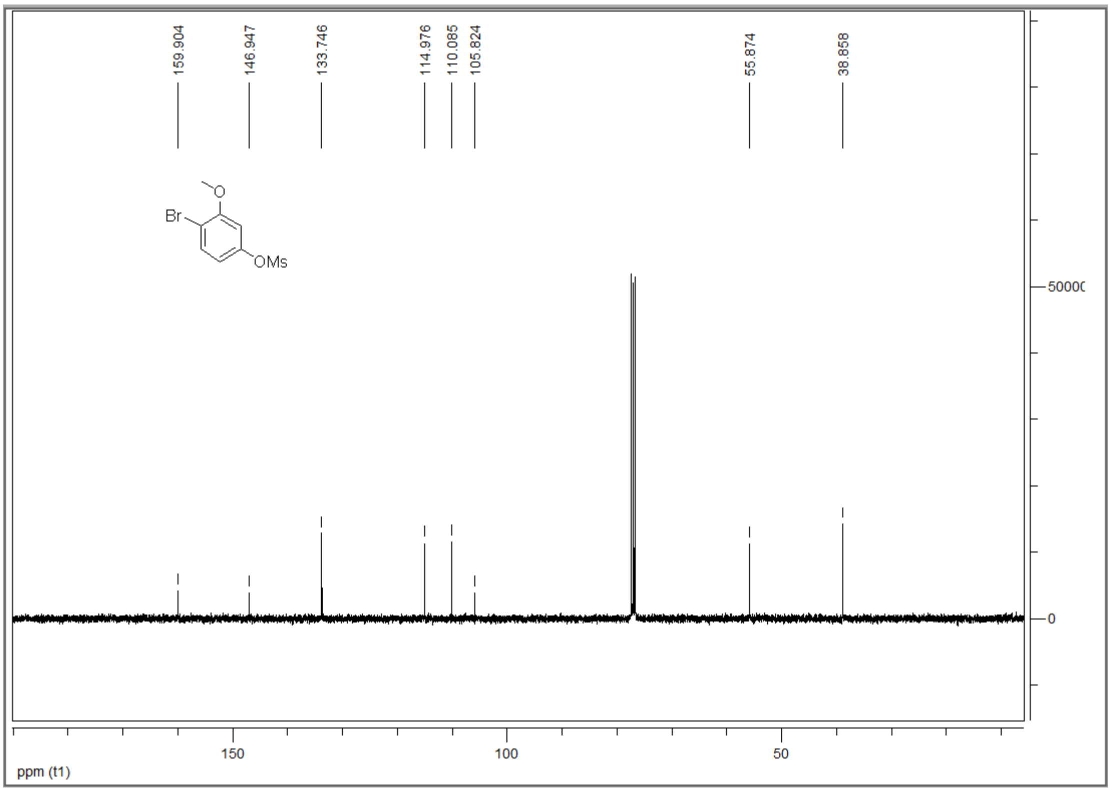

159.904
146.947
133.746
114.976
110.085
105.824
55.874
38.858
Br
O
OMs
50000
0
150
100
50
ppm (t1)

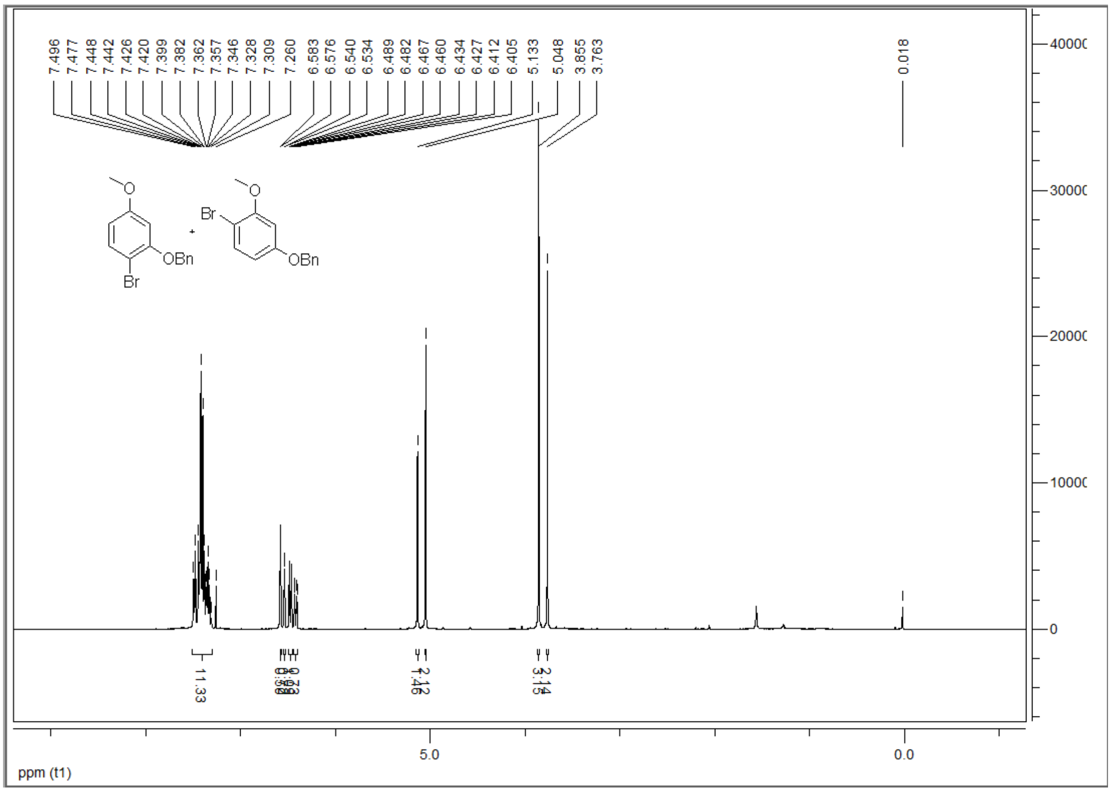
7.496
7.477
7.448
7.442
7.426
7.420
7.399
7.382
7.362
7.357
7.346
7.328
7.309
7.260
6.583
6.576
6.540
6.534
6.489
6.482
6.467
6.460
6.434
6.427
6.412
6.405
5.133
5.048
3.855
3.763
0.018
Br
OBn
OBn
Br
11.33
2.12
2.14
5.0
0.0
ppm (t1)

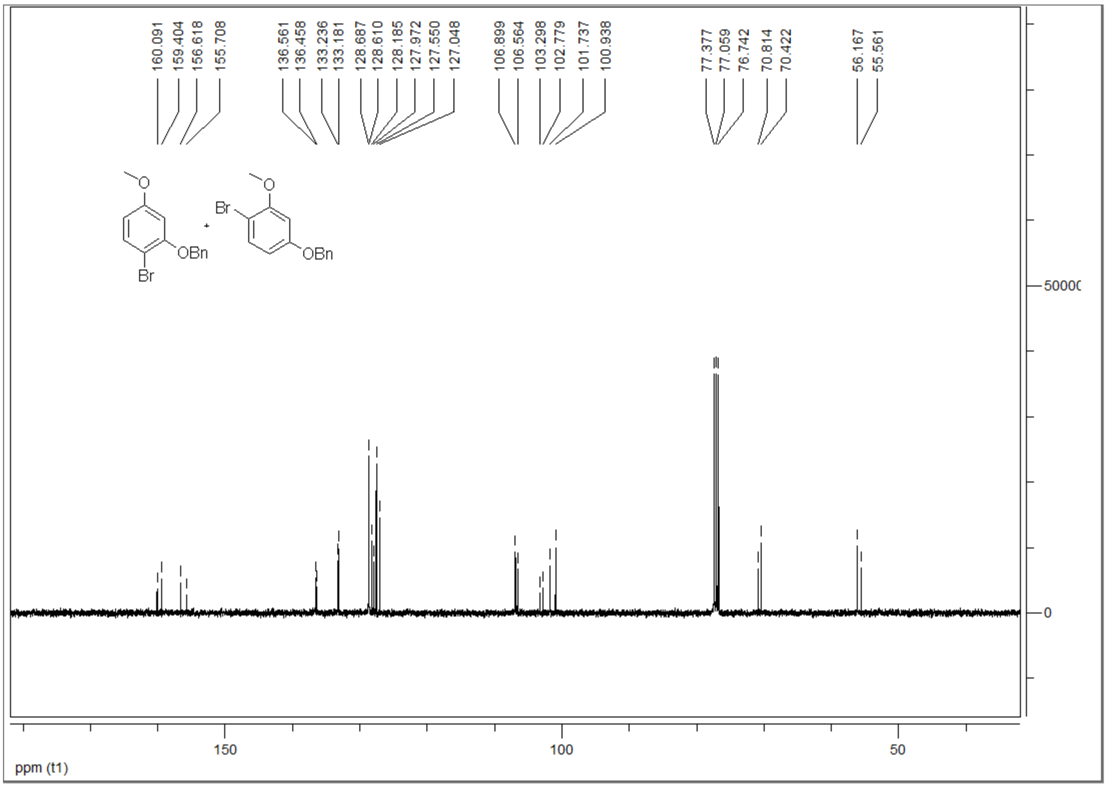
160.091
159.404
156.618
155.708
136.561
136.458
133.236
133.181
128.687
128.610
128.185
127.972
127.550
127.048
106.899
106.564
103.298
102.779
101.737
100.938
77.377
77.059
76.742
70.814
70.422
56.167
55.561
Br
OBn
Br
OBn
50000
0
150
100
50
ppm (t1)

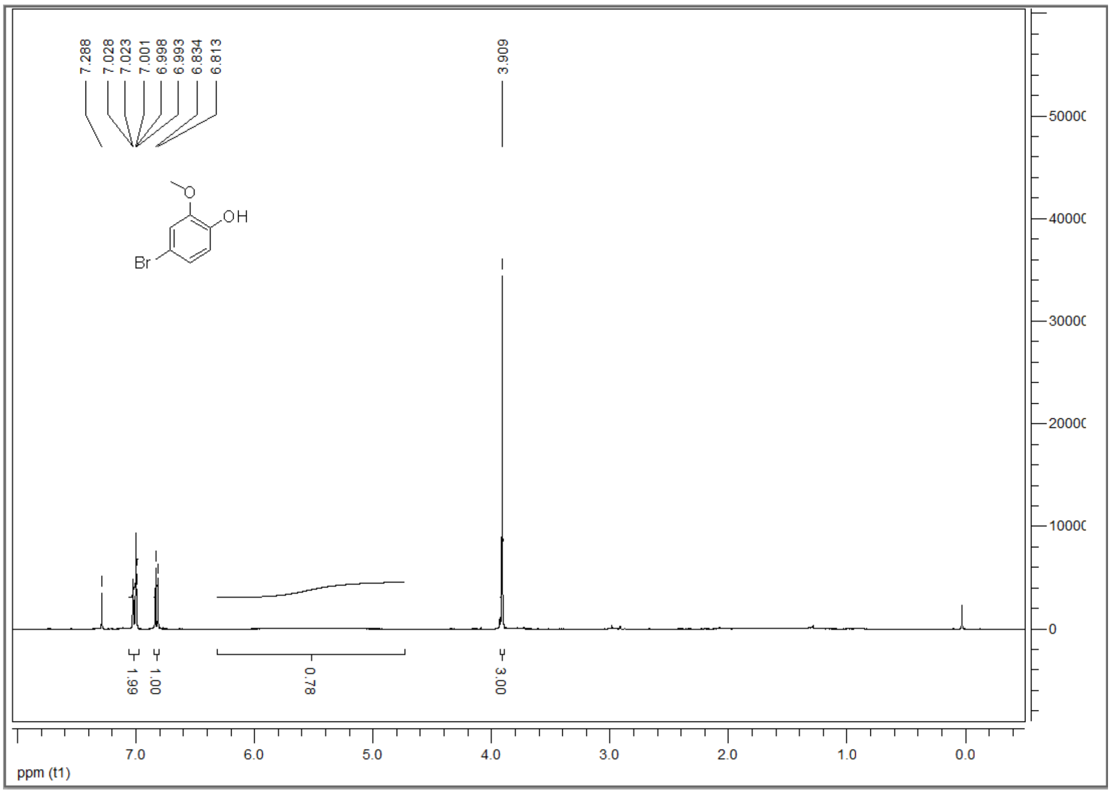
7.288
7.028
7.023
7.001
6.998
6.993
6.834
6.813
3.909
OH
Br
O
1.99
1.00
0.78
3.00
ppm (t1)

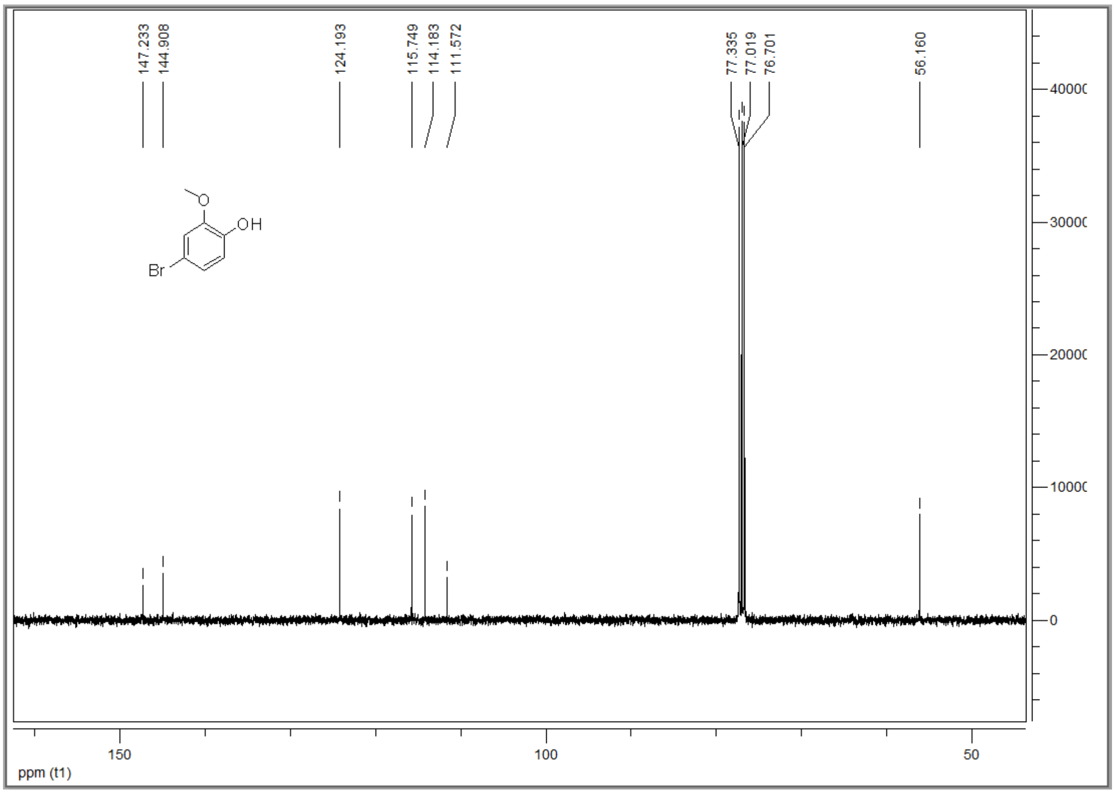
147.233
144.908
124.193
115.749
114.183
111.572
77.335
77.019
76.701
56.160
OH
Br
O
ppm (t1)

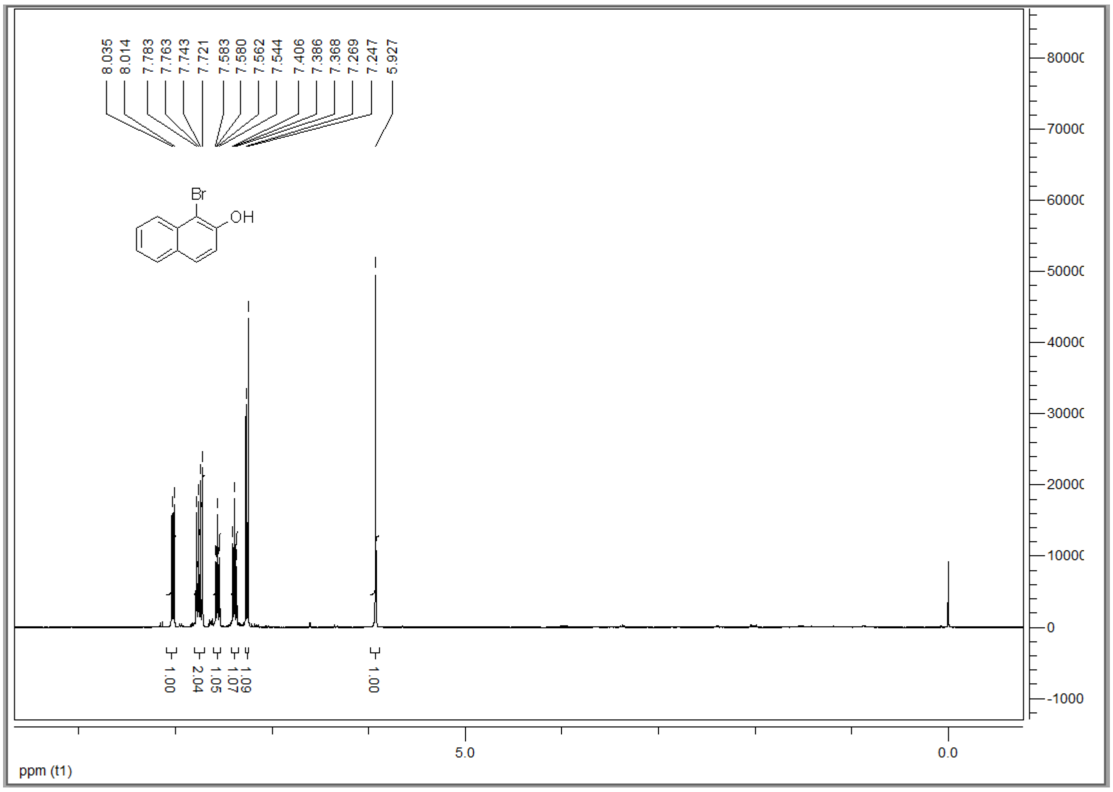
8.035
8.014
7.783
7.763
7.743
7.721
7.583
7.580
7.562
7.544
7.406
7.386
7.368
7.269
7.247
5.927
Br
OH
1.00
2.04
1.05
1.07
1.09
1.00
5.0
0.0
ppm (t1)

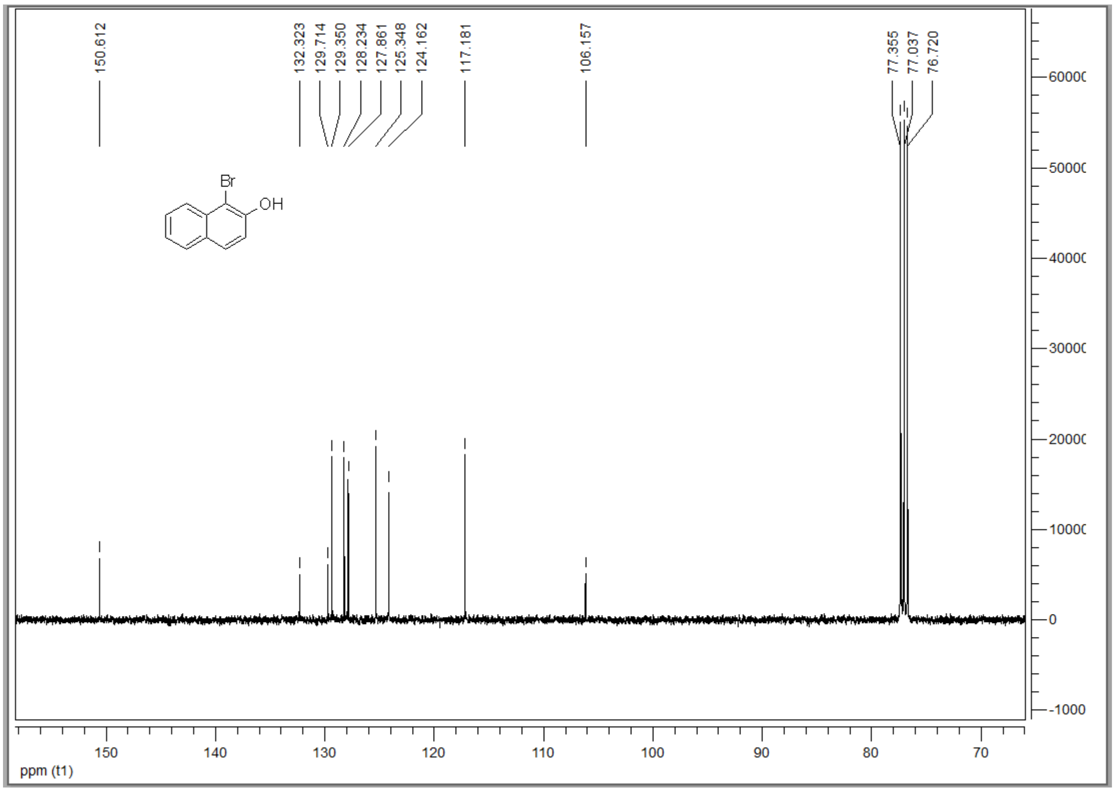
150.612
132.323
129.714
129.350
128.234
127.861
125.348
124.162
117.181
106.157
77.355
77.037
76.720
Br
OH
150
140
130
120
110
100
90
80
70
ppm (t1)

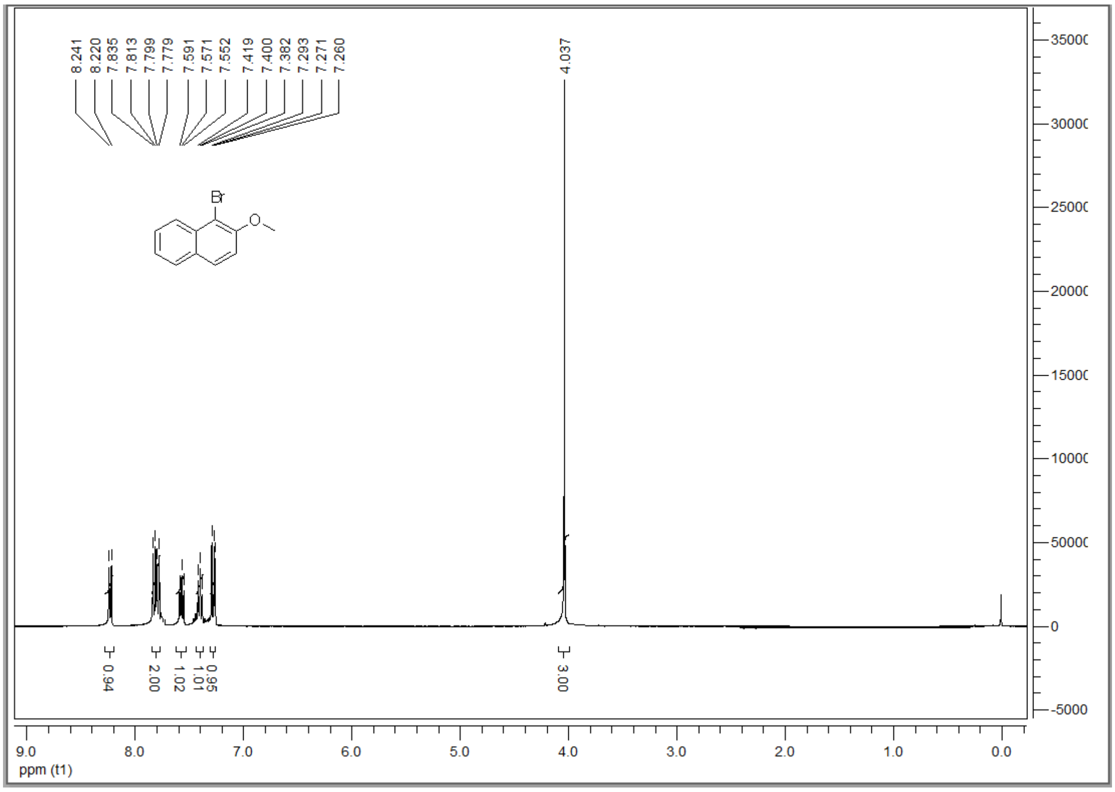
8.241
8.220
7.835
7.813
7.799
7.779
7.591
7.571
7.552
7.419
7.400
7.382
7.293
7.271
7.260
4.037
Br
O
0.94
2.00
1.02
1.01
0.95
3.00
35000
30000
25000
20000
15000
10000
5000
0
-5000
9.0
8.0
7.0
6.0
5.0
4.0
3.0
2.0
1.0
0.0
ppm (t1)

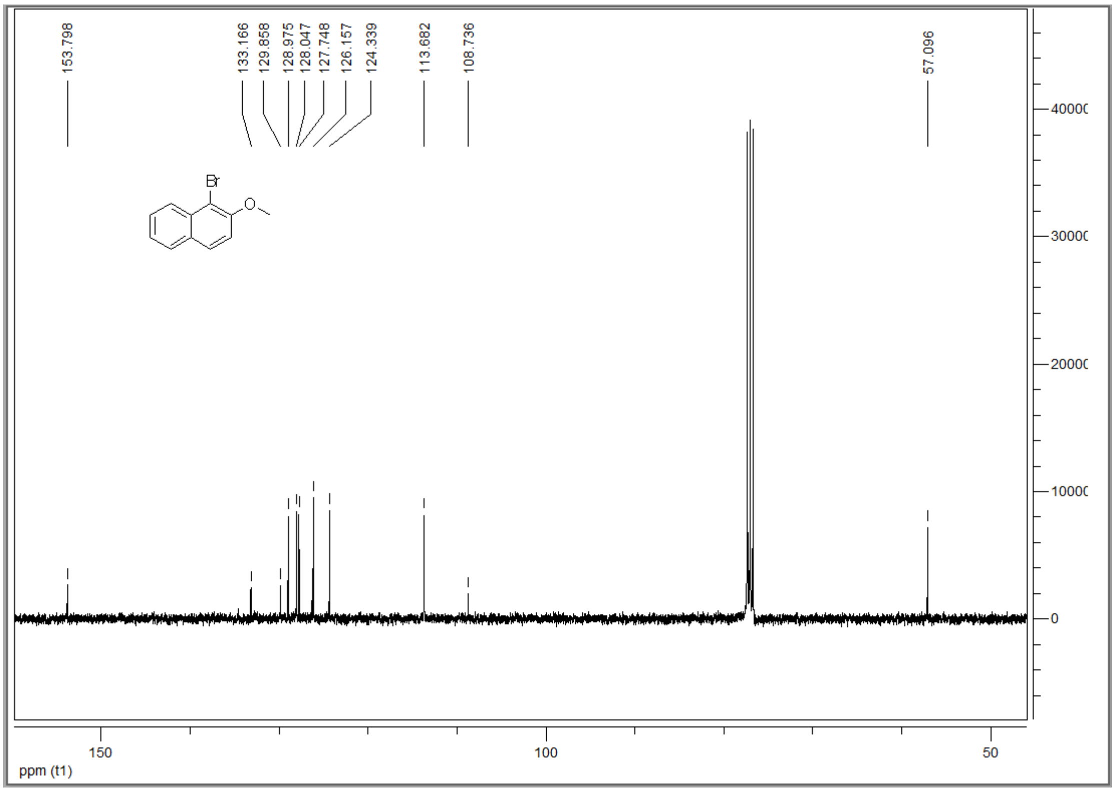
153.798
133.166
129.858
128.975
128.047
127.748
126.157
124.339
113.682
108.736
57.096
Br
O
40000
30000
20000
10000
0
150
100
50
ppm (t1)

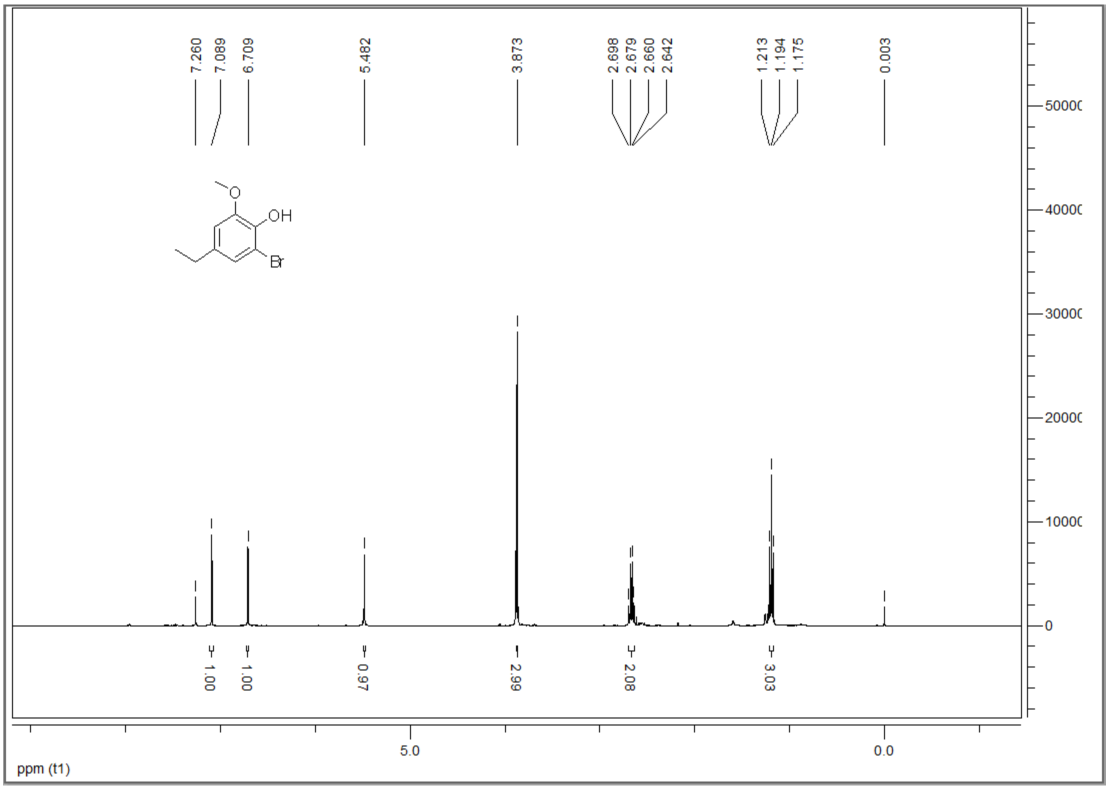
7.260
7.089
6.709
5.482
3.873
2.698
2.679
2.660
2.642
1.213
1.194
1.175
0.003
OH
Br
1.00
1.00
0.97
2.99
2.08
3.03
5.0
0.0
ppm (t1)

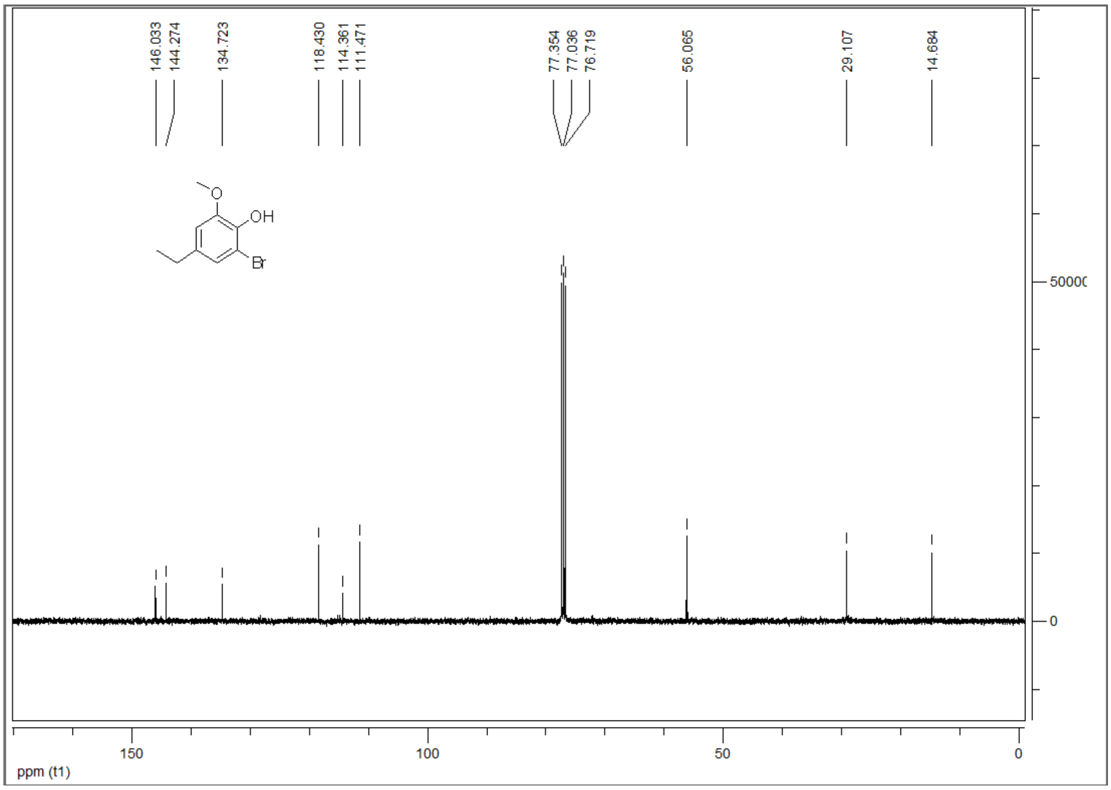
146.033
144.274
134.723
118.430
114.361
111.471
77.354
77.036
76.719
56.065
29.107
14.684
OH
Br
150
100
50
0
ppm (t1)

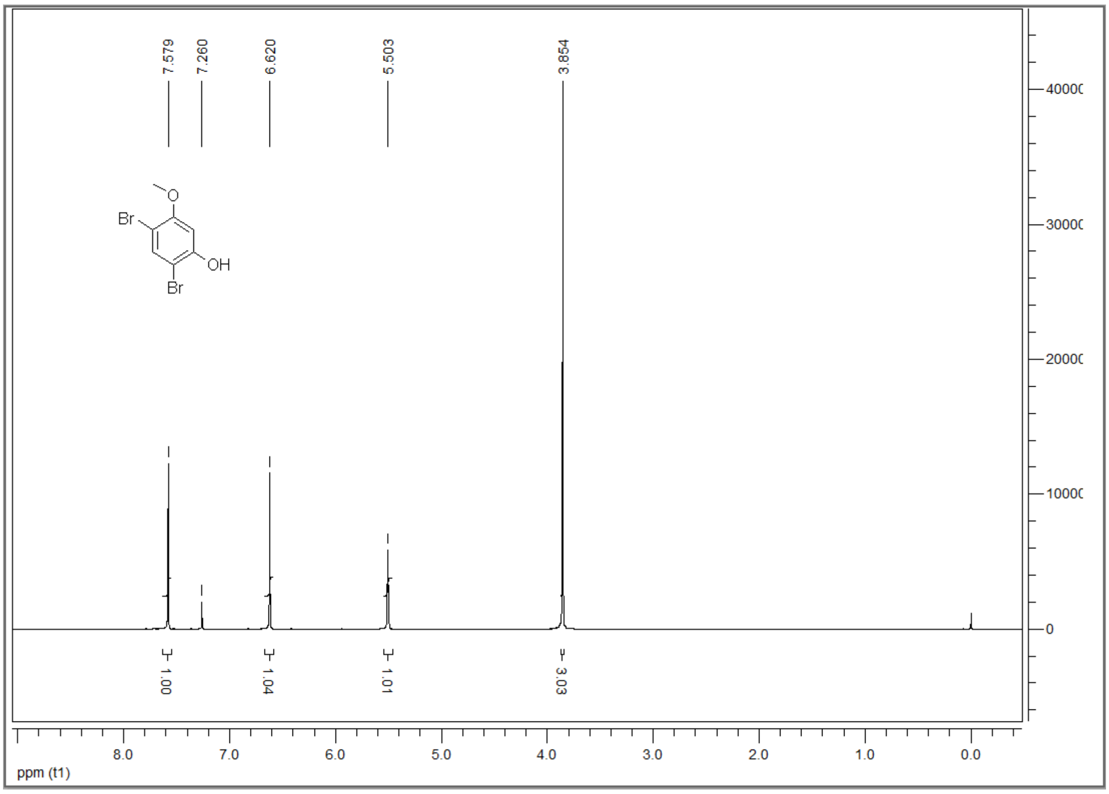
7.579
7.260
6.620
5.503
3.854
Br
O
OH
Br
1.00
1.04
1.01
3.03
ppm (t1)
8.0
7.0
6.0
5.0
4.0
3.0
2.0
1.0
0.0
40000
30000
20000
10000
0

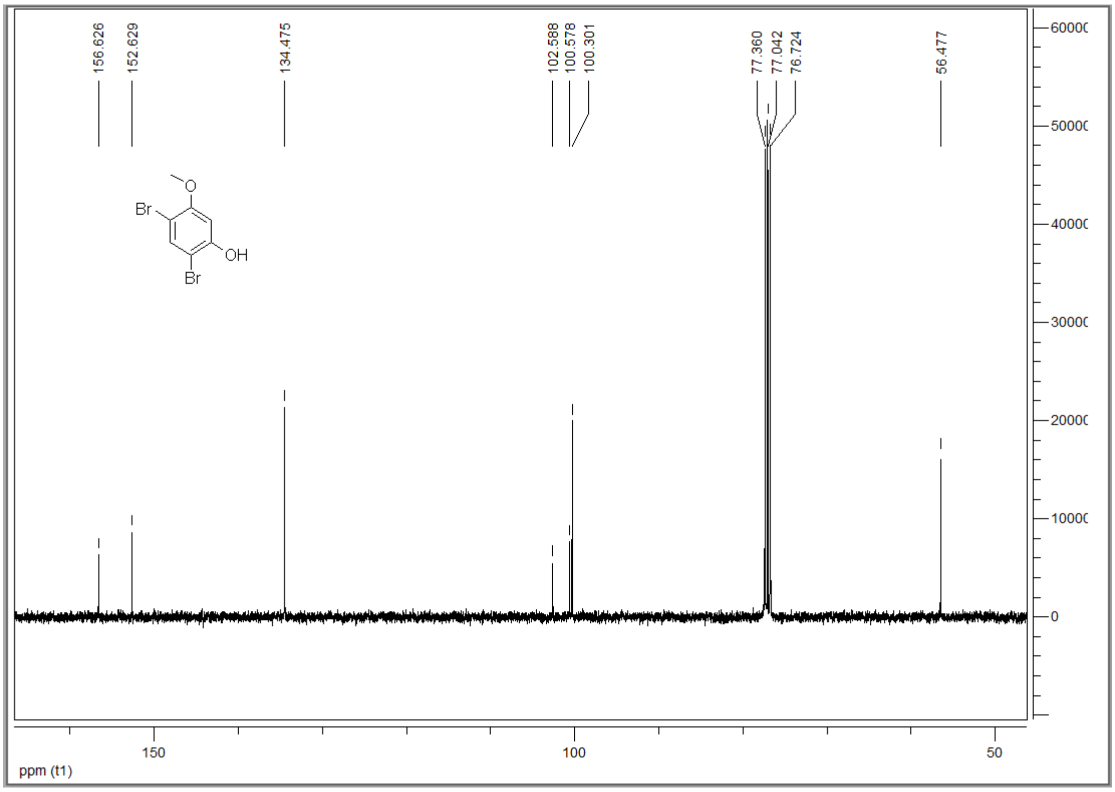
156.626
152.629
134.475
102.588
100.578
100.301
77.360
77.042
76.724
56.477
Br
O
OH
Br
ppm (t1)
150
100
50
60000
50000
40000
30000
20000
10000
0

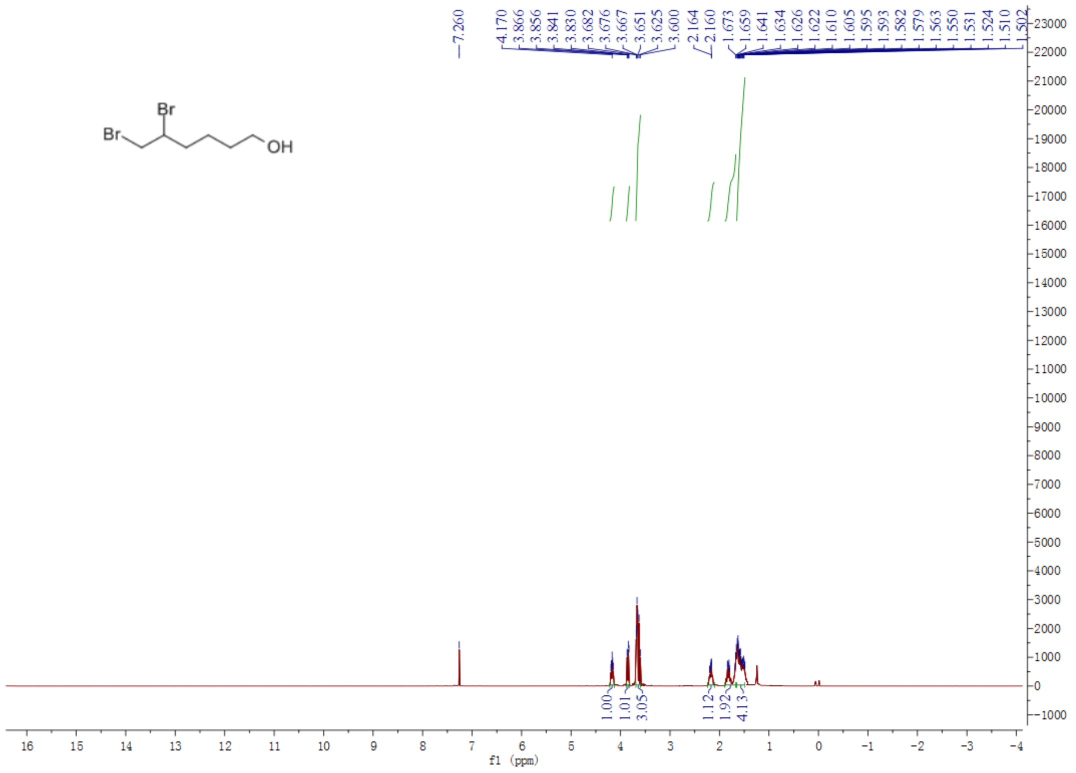

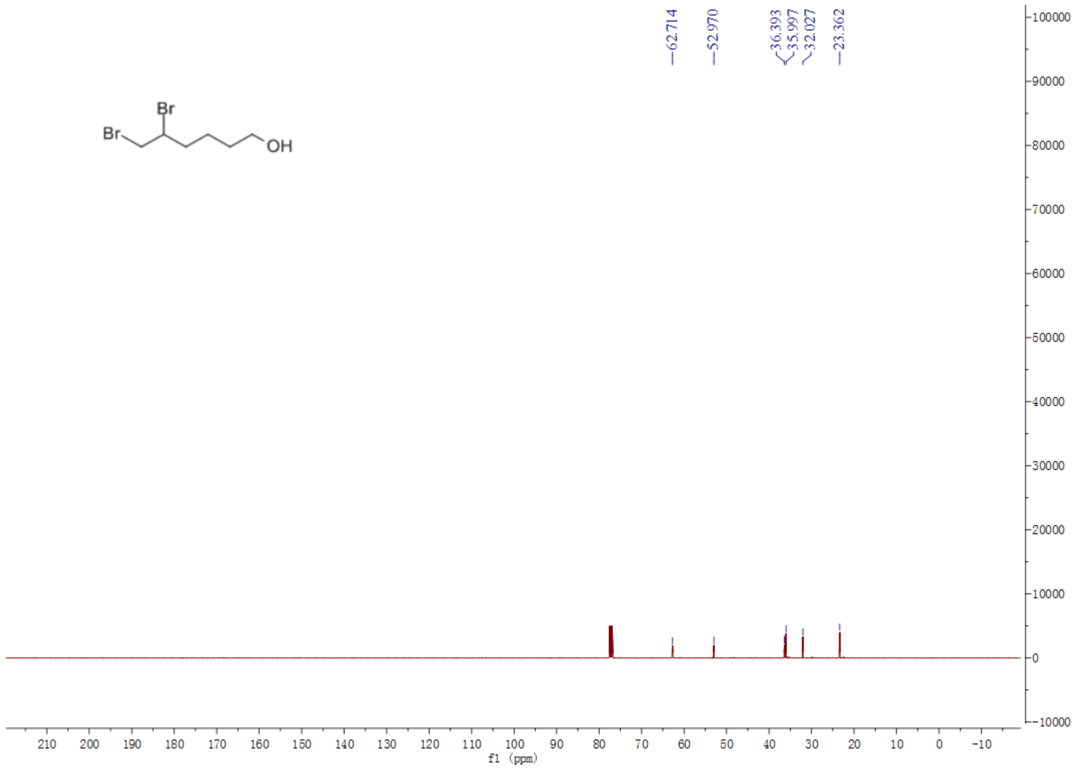

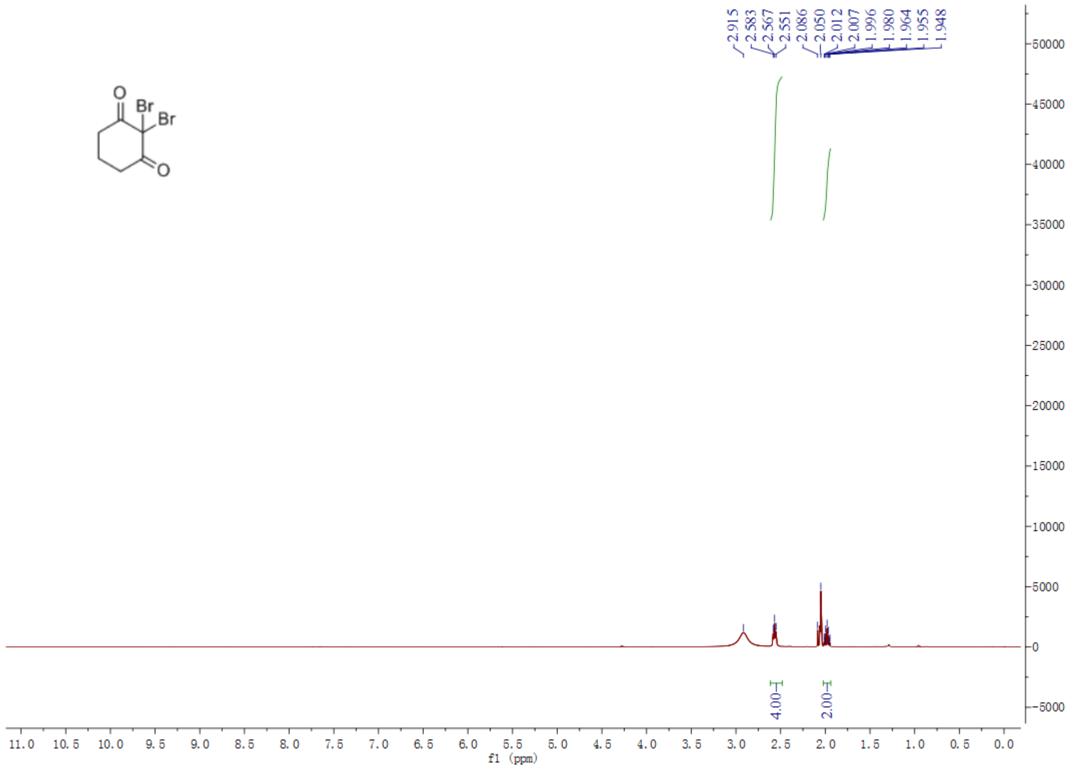
Br
Br
O
O
2.915
2.583
2.567
2.551
2.086
2.050
2.012
2.007
1.996
1.980
1.964
1.955
1.948
4.00
2.00
f1 (ppm)

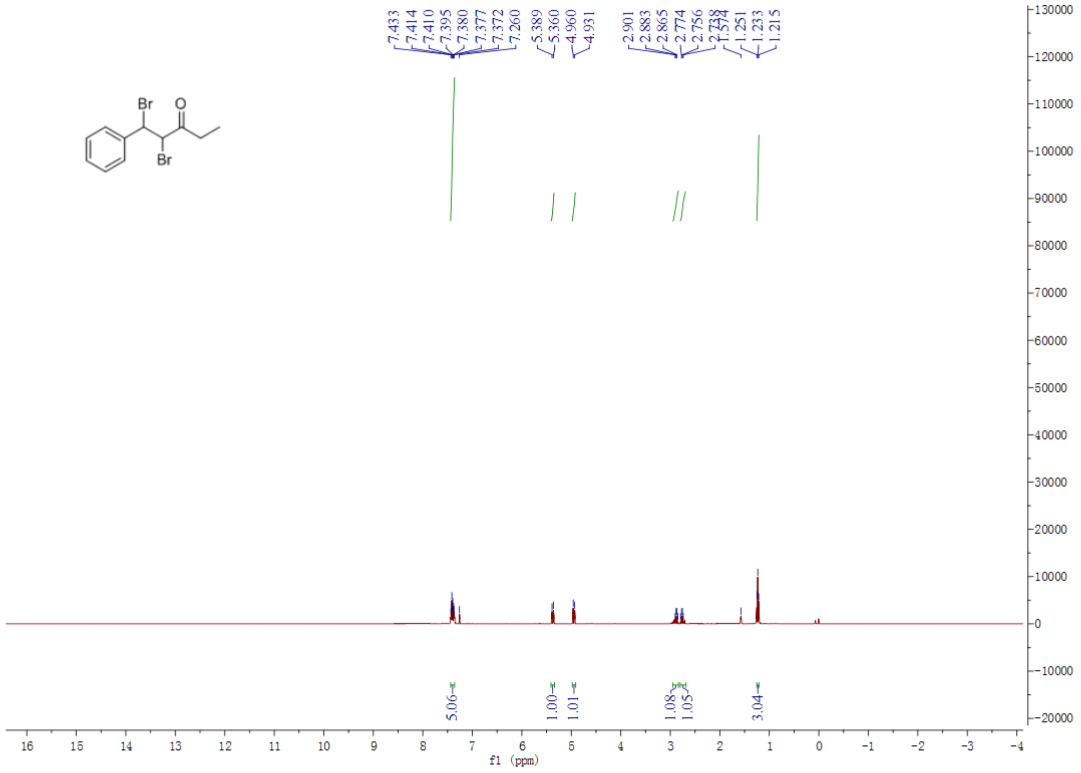

7.433
7.414
7.410
7.395
7.380
7.377
7.372
7.260
5.389
5.360
4.960
4.931
2.901
2.883
2.865
2.774
2.756
2.738
1.574
1.251
1.233
1.215
Br
O
Br
5.06
1.00
1.01
1.08
1.05
3.04
f1 (ppm)

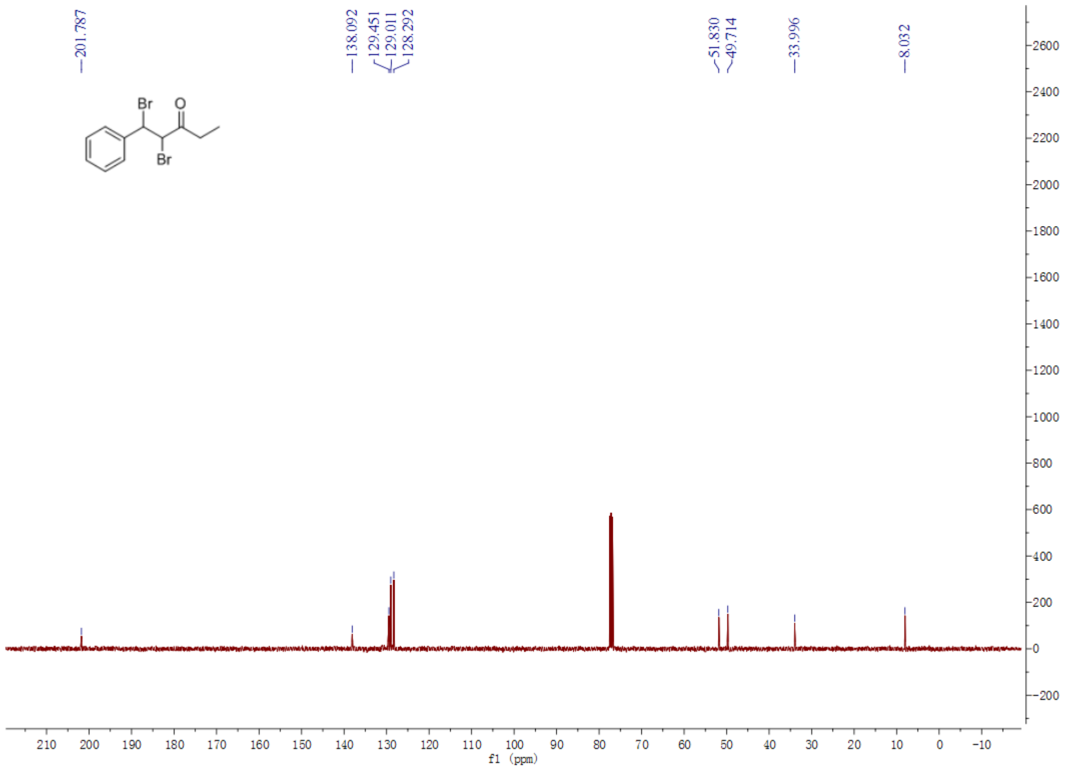

201.787
138.092
129.451
129.011
128.292
51.830
49.714
33.996
8.032
Br
O
Br
f1 (ppm)

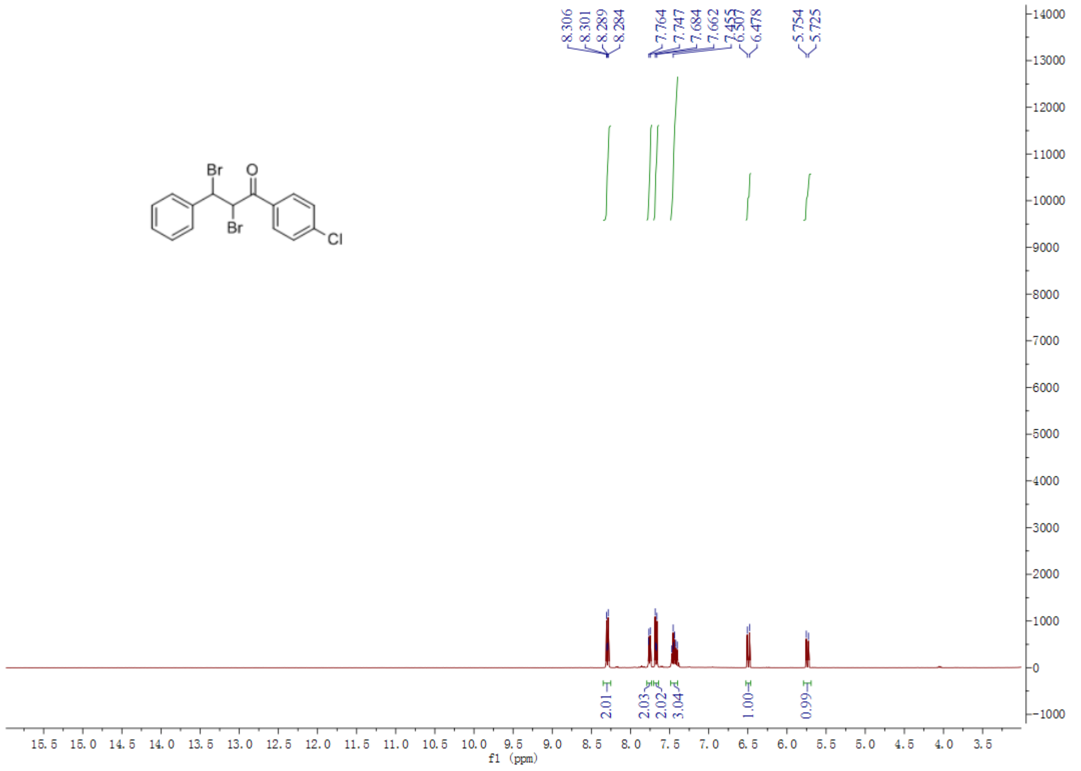

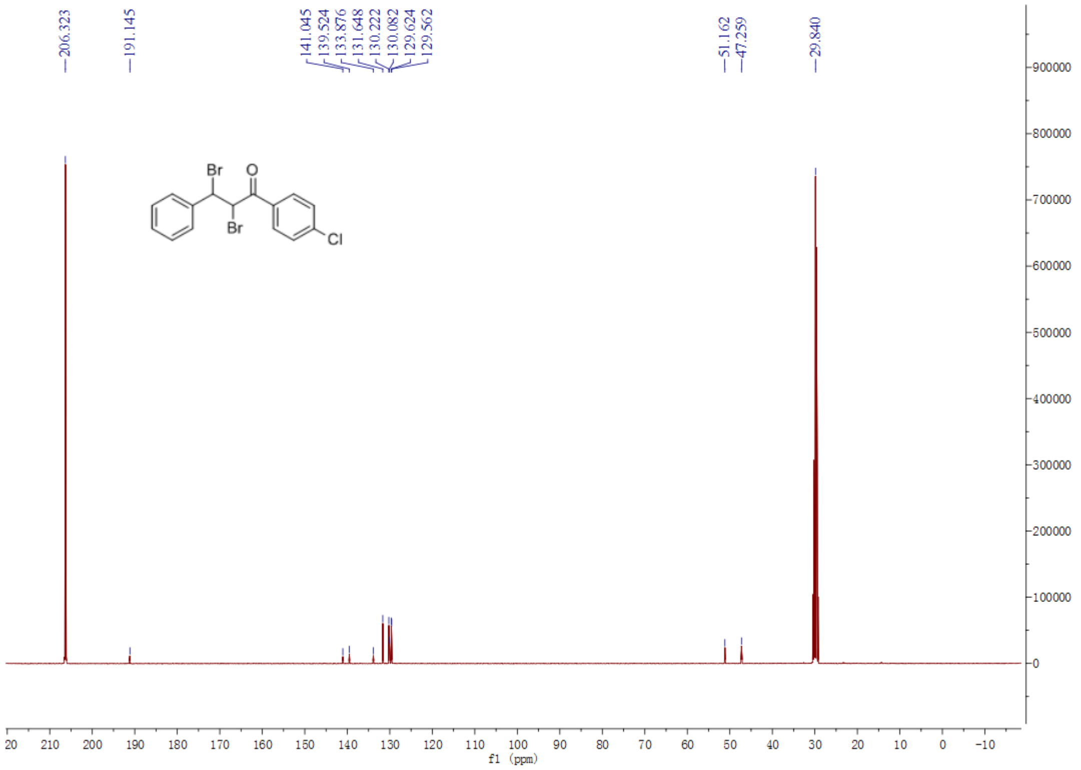

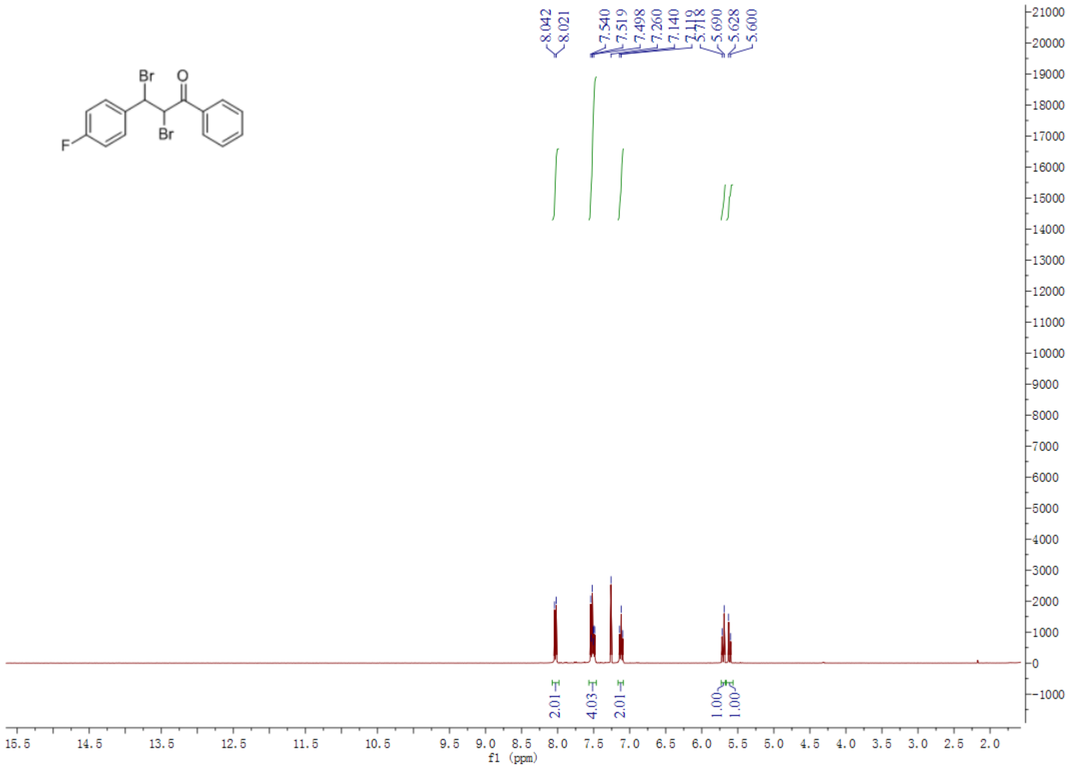
Br
O
F
Br
8.042
8.021
7.540
7.519
7.498
7.260
7.140
7.119
5.718
5.690
5.628
5.600
2.01
4.03
2.01
1.00
1.00
f1 (ppm)

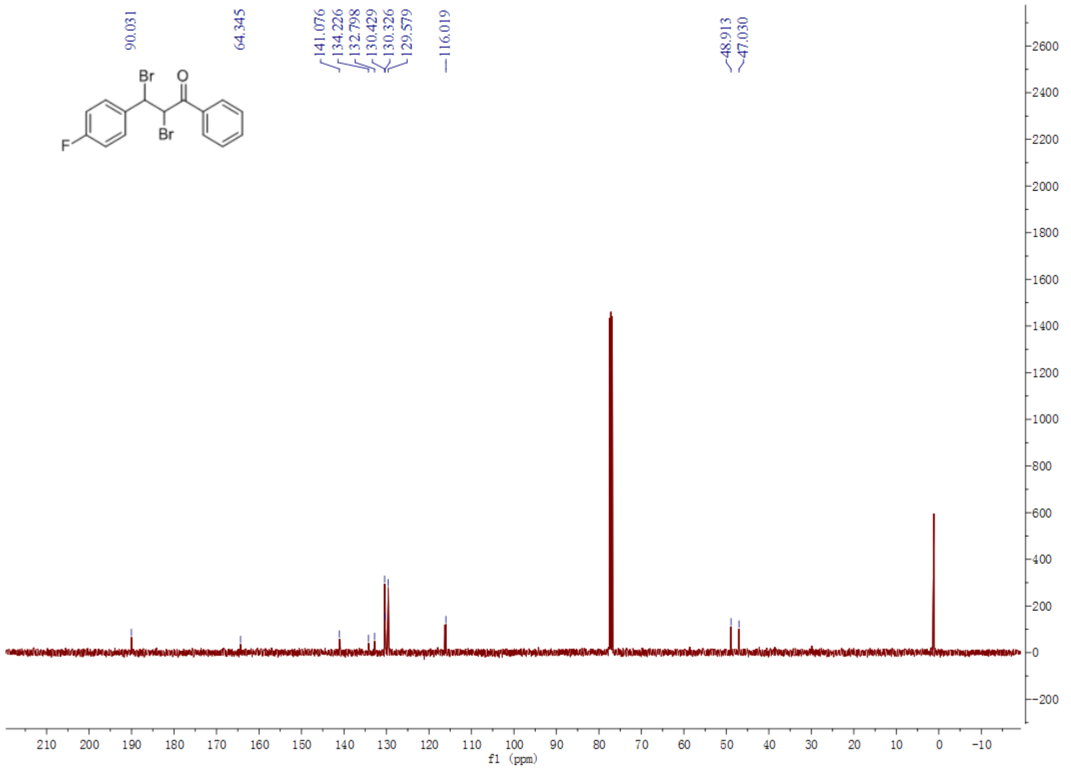
90.031
64.345
141.076
134.226
132.798
130.429
130.326
129.579
116.019
48.913
47.030
Br
O
F
Br
f1 (ppm)

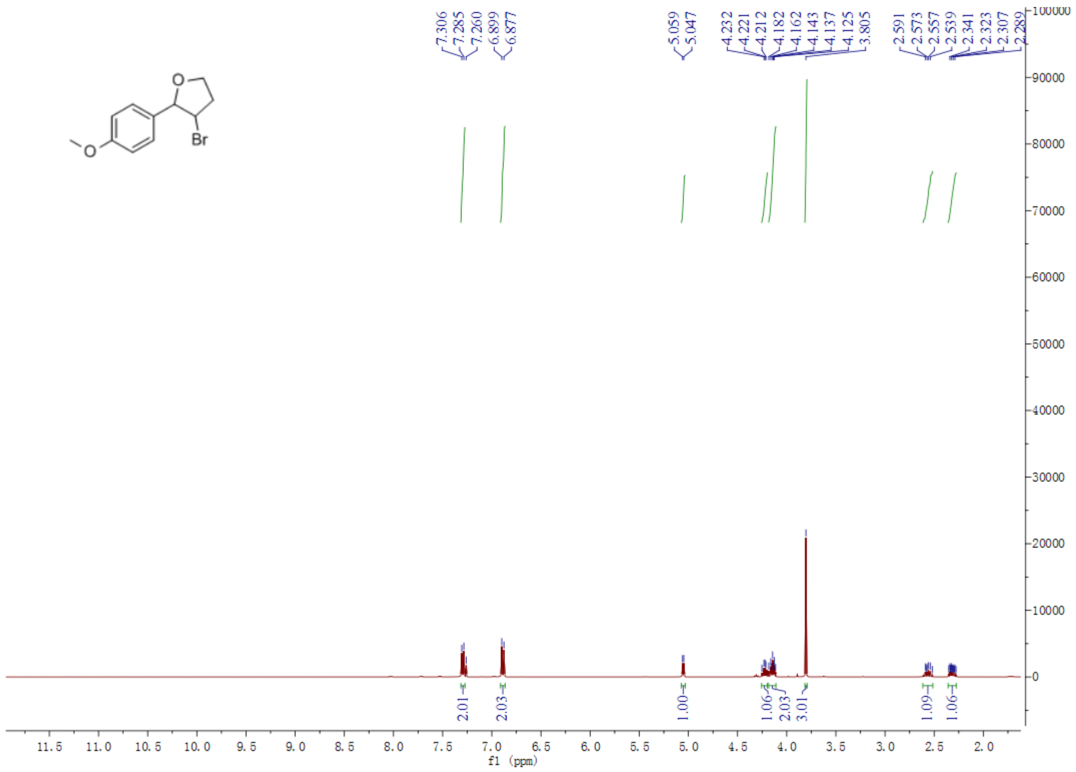

O
Br
O
7.306
7.285
7.260
6.899
6.877
5.059
5.047
4.232
4.221
4.212
4.182
4.162
4.143
4.137
4.125
3.805
2.591
2.573
2.557
2.539
2.341
2.323
2.307
2.289
2.01
2.03
1.00
1.06
2.03
3.01
1.09
1.06
f1 (ppm)

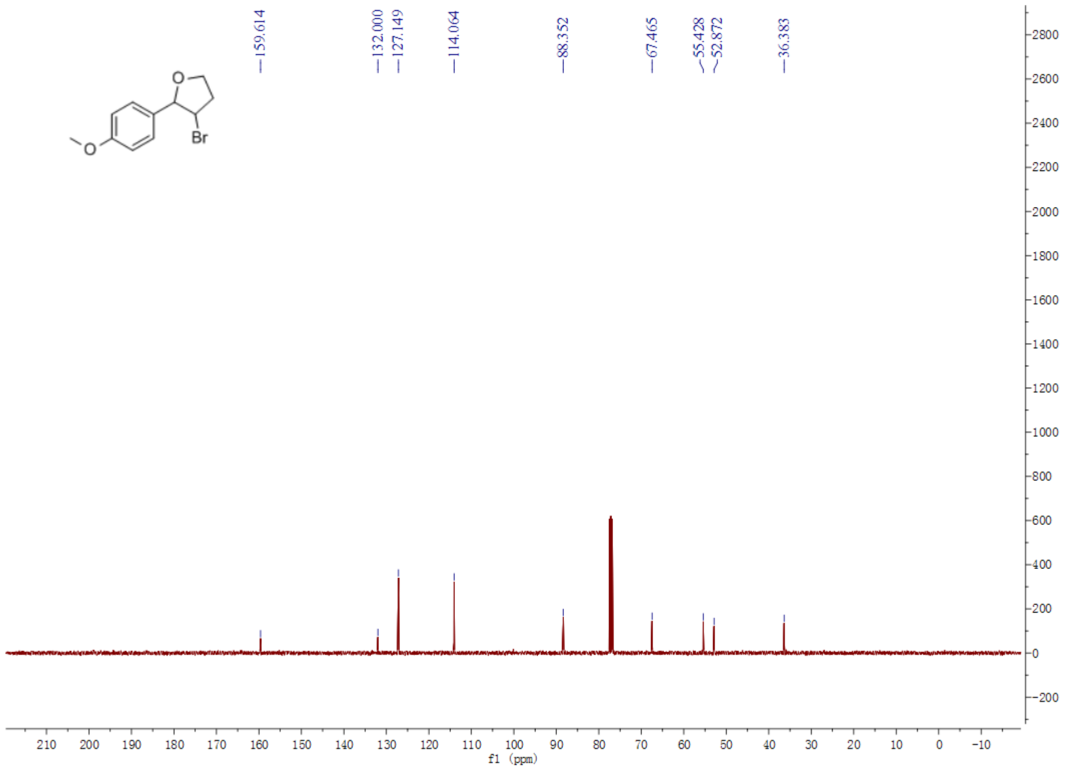

O
Br
O
159.614
132.000
127.149
114.064
88.352
67.465
55.428
52.872
36.383
f1 (ppm)

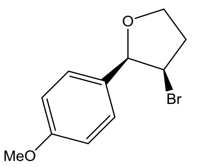
O
Br
MeO

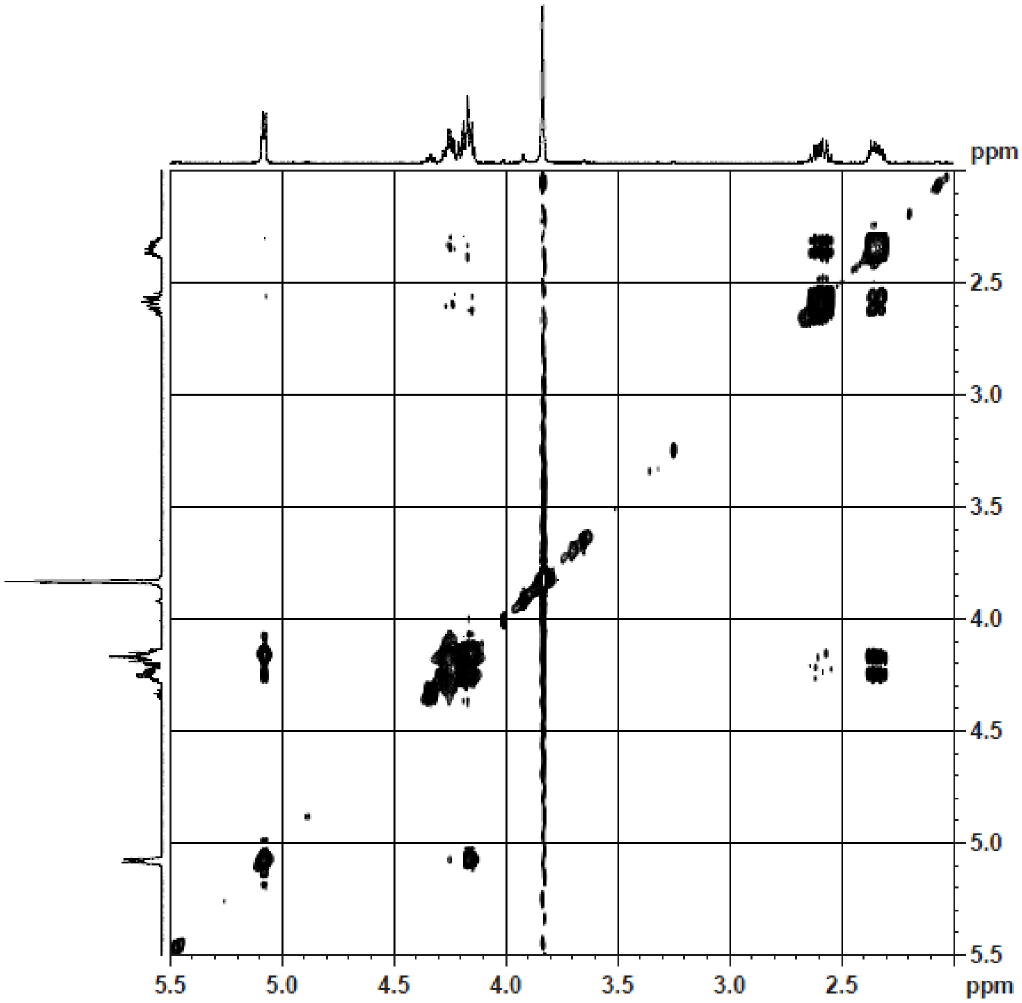
ppm
2.5
3.0
3.5
4.0
4.5
5.0
5.5
5.5
5.0
4.5
4.0
3.5
3.0
2.5
ppm

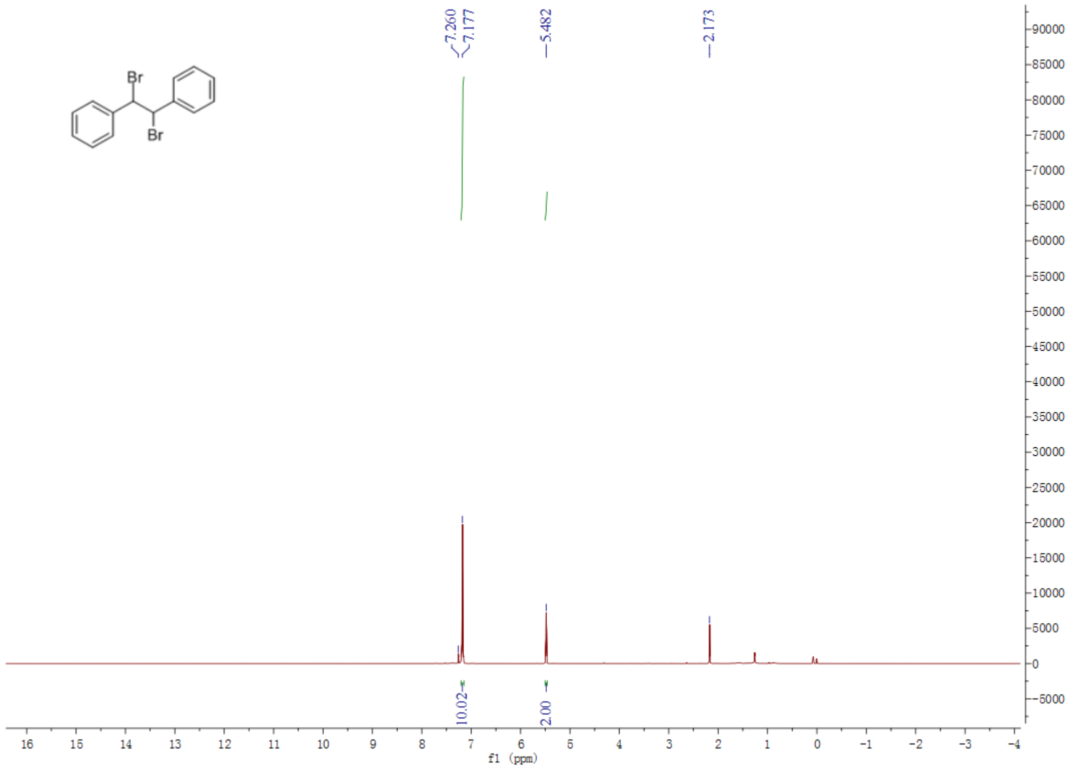

Br
Br
7.260
7.177
5.482
2.173
10.02
2.00
f1 (ppm)

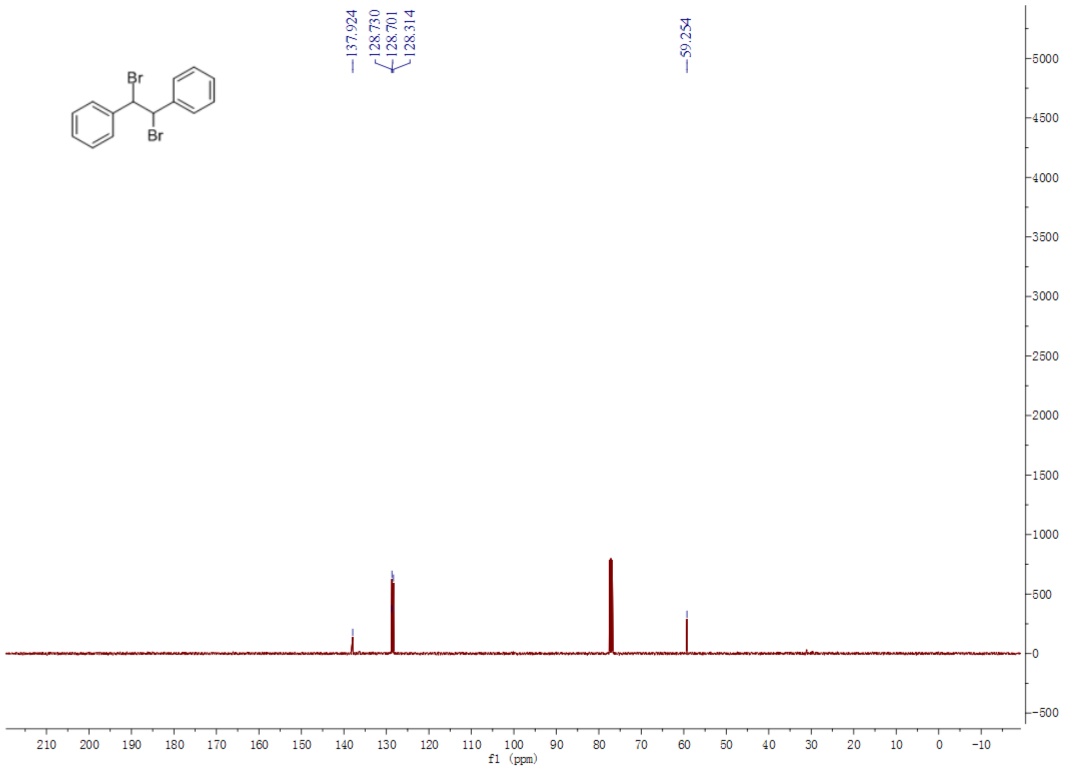

Br
Br
137.924
128.730
128.701
128.314
59.254
f1 (ppm)

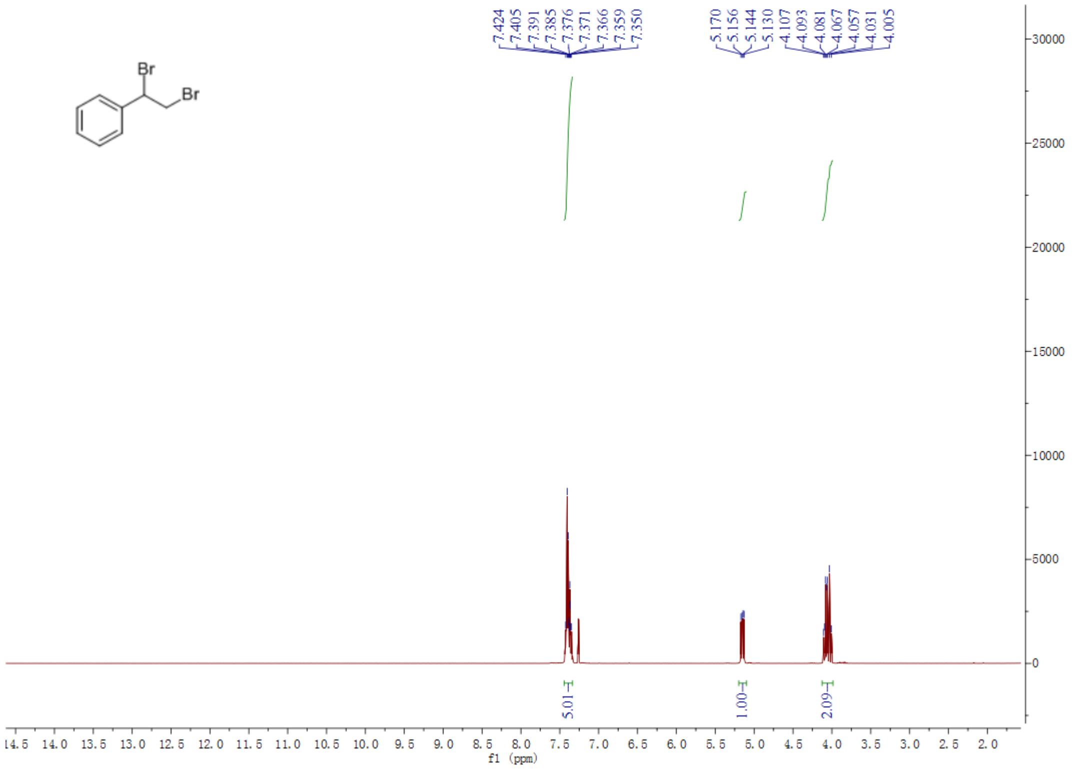
Br
Br
7.424
7.405
7.391
7.385
7.376
7.371
7.366
7.359
7.350
5.170
5.156
5.144
5.130
4.107
4.093
4.081
4.067
4.057
4.031
4.005
5.01
1.00
2.09
f1 (ppm)

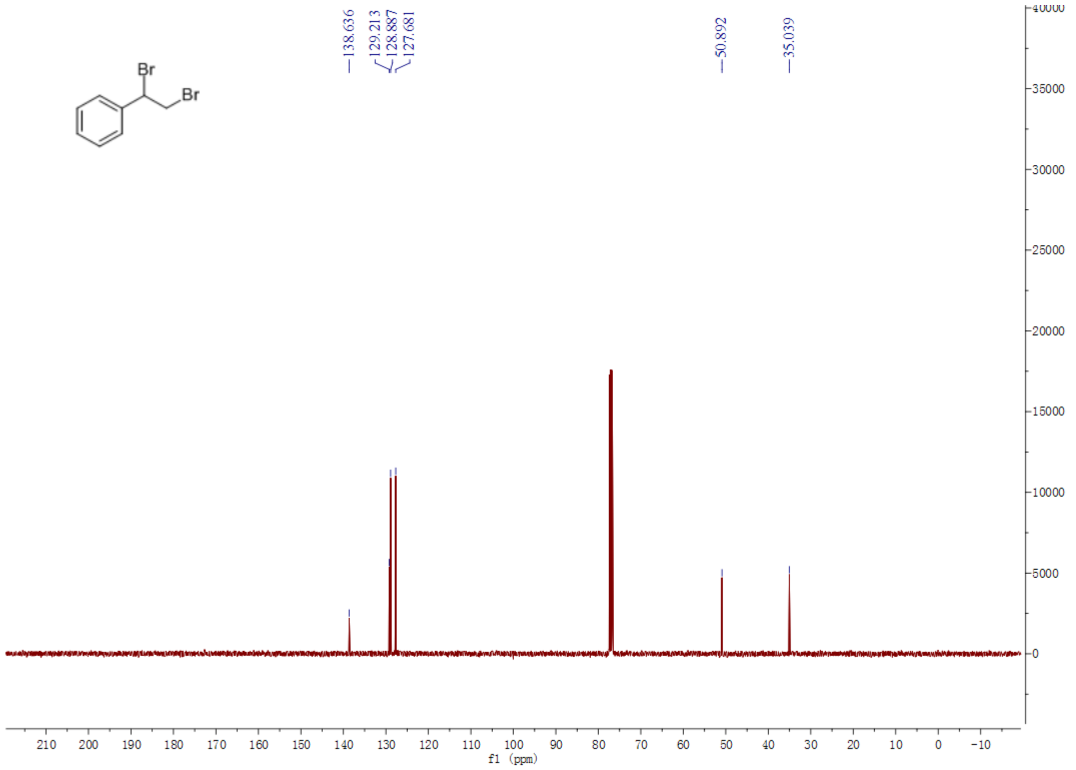
Br
Br
138.636
129.213
128.887
127.681
50.892
35.039
f1 (ppm)
